# Anti-cancer organoruthenium(ii) complexes and their interactions with cysteine and its analogues. A mass-spectrometric study†

**DOI:** 10.1039/c8dt04350g

**Published:** 2019-01-28

**Authors:** Anamarija Briš, Juraj Jašík, Iztok Turel, Jana Roithová

**Affiliations:** Department of Organic Chemistry, Faculty of Science, Charles University Hlavova 2030/8 128 43 Prague Czech Republic; Ruđer Bošković Institute Bijenička 54 10 000 Zagreb Croatia; Faculty of Chemistry and Chemical Technology, University of Ljubljana Večna pot 113 SI-1000 Ljubljana Slovenia; Institute for Molecules and Materials, Radboud University Heyendaalseweg 135 6525 AJ Nijmegen The Netherlands jana.roithova@ru.nl

## Abstract

The ruthenium complexes [Ru(CYM)(*p*-Cl-dkt)(Cl)] (**1**), [Ru(CYM)(pta)(*p*-Cl-dkt)]PF_6_ (**2**), and [Ru(CYM)(pta)Cl_2_] (**3**, RAPTA-C) (CYM = *para*-cymene, *p*-Cl-dkt = 1-(4-chlorophenyl)-4,4,4-trifluorobutane-1,3-dione, pta = 1,3,5-triaza-7-phosphaadamantane) are biologically active and show anti-cancer activities, albeit with different mechanisms. To further understand these mechanisms, we compared their speciation in aqueous solutions with an amino acid (cysteine), with an amino acid derivative (*N*-acetylcysteine) and with a tripeptide (glutathione) by Mass Spectrometry (MS). Here, we show that all ruthenium complexes have high selectivity for cysteine and cysteine-derived molecules. On one hand, [Ru(CYM)(*p*-Cl-dkt)(Cl)] undergoes solvolysis in water and forms [Ru_2_(CYM)_2_(OH)_3_]^+^. Subsequently, all hydroxyl anions are exchanged by deprotonated cysteine. Infrared Photodissociation Spectroscopy (IRPD) showed that cysteine binds to the ruthenium atoms *via* the deprotonated thiol group and that sulfur bridges the ruthenium centers. On the other hand, the pta-bearing complexes remain monometallic and undergo only slow Cl or *p*-Cl-dkt exchange by deprotonated cysteine. Therefore, the pta ligand protects the ruthenium complexes from ligand exchange with water and from the formation of biruthenium clusters, possibly explaining why the mechanism of pta-bearing ruthenium complexes is not based on ROS production but on their reactivity as monometallic complexes.

## Abstract 2

ESI-MS study of ruthenium complexes shows their high selectivity toward thiol containing molecules and formation of larger thiolate-bound clusters in absence of a protecting ligand such as pta.

## Graphical abstract

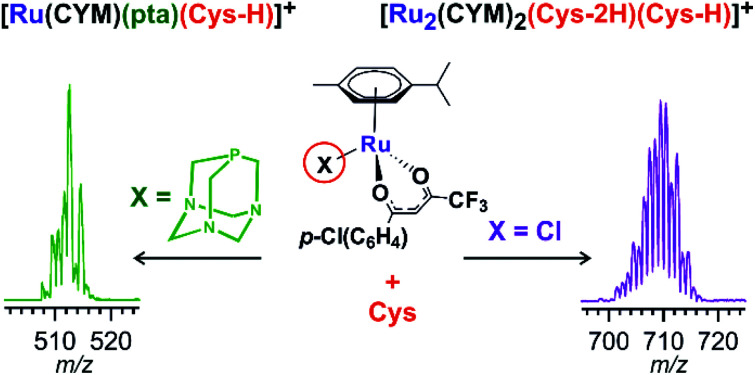

## Introduction

Ruthenium complexes are popular in synthetic chemistry because they can catalyse important reactions, including C–H activations or C–C couplings.^1^ In addition to synthetic applications, ruthenium complexes have shown interesting biological activities such as anti-cancer effects.^2,3^ Therefore, the design of organometallic ruthenium complexes for possible medicinal use has become a hot research topic in recent years.^4^

Drug design is usually driven by the mode of action of the drug.^5,6^ Previous studies have shown that some complexes produce reactive oxygen species in the cell (a), thereby inducing apoptosis.^7^ Other complexes have a different mode of action, presumably involving interaction with DNA (b)^8,9^ or proteins (c),^9,10^ with cytostatic effects. The mode of action (a, b or c) of ruthenium complexes most likely depends on its stability in the biological environment and speciation. Here, we will present a comparative study of three, rather similar, ruthenium complexes (Table 1), albeit with different biological activities.

**Table 1:** Ruthenium(ii) complexes investigated by ESI-MS and their biological activity

Complex	**1**	**2**	**3**
Notation	[Ru(CYM)(*p*-Cl-dkt)Cl]	[Ru(CYM)(pta)(*p*-Cl-dkt)]PF_6_	[Ru(CYM)(pta)Cl_2_] (RAPTA-C)
Formula	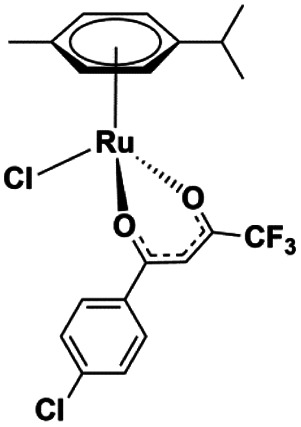	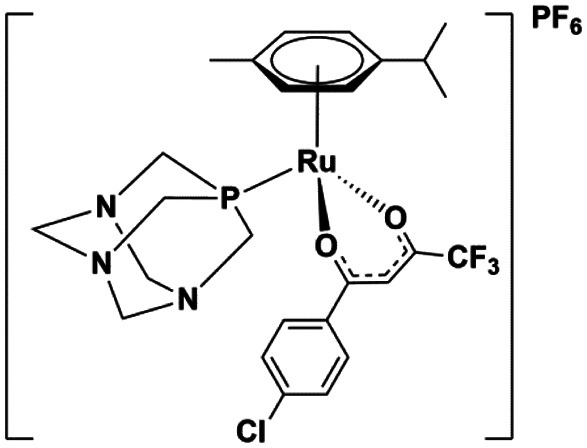	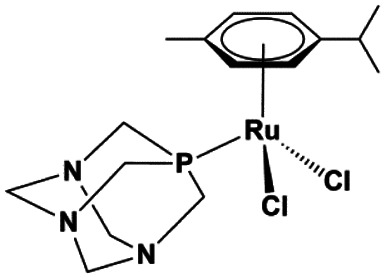
IC_50_ [μM] ovarian cell	17	8	65 ± 15 (ref. 13)
IC_50_ [μM] osteosarcoma	64	41	
ROS production	Yes	No	No
Cell uptake Jurkat cells^a^	1.5 × 10^−4^ ng	1 × 10^−4^ ng	5 × 10^−5^ ng

a Concentration of **1–3** was 10 μM. See ref. 2 for more details.

All complexes studied herein have a ruthenium(ii)-*para*-cymene (CYM) core. In addition, the first complex has two different anionic ligands, chloride and *β*-dikenonate *p*-Cl-dkt (*p*-Cl-dkt = 1-(4-chlorophenyl)-4,4,4-trifluorobutane-1,3-dione); *β*-dikenonates being established building blocks in anticancer drug design.^11^ The second complex has a bulky neutral pta ligand instead of chloride (pta = 1,3,5-triaza-7-phosphaadamantane; see Table 1). Thus, its charge is compensated by the PF_6_^−^ counter ion. The last complex is, thus far, the most promising anti-cancer drug candidate, termed RAPTA-C. This complex has a pta ligand and two chlorido ligands.

It is known that the first complex (**1**) produces radical oxygen species (ROS) in the cell environment and induces apoptosis.^2^ ROS production by **1** could be counteracted by *N*-acetylcysteine (NAC) addition, a radical scavenger. Pta-bearing complexes produce no ROS. However, both complexes, **2** and **3**, are potent cytostatic drugs. The difference in the biological activity between **1** on one hand and **2** and **3** on the other is correlated with the presence of the pta ligand, as previously shown in a larger set of ruthenium complexes.^2^

Another interesting difference in the biological activity of **1**, **2**, and **3** was found when decreasing the cellular levels of glutathione, a natural radical scavenger, thus, enabling a more drastic effect of ROS. Indeed, the anti-cancer activity of **1** increased approximately 4 times under such conditions. Surprisingly, the anti-cancer activity of **2** also increased approximately 4 times. Even more surprisingly, however, the anti-cancer activity of RAPTA-C (**3**) increased more than 25 times. Hence, the decrease in glutathione levels not only decreases ROS scavenging but also stimulates pta-bearing complexes, which produce no ROS.

All these differences and similarities raise questions about the effect of the pta ligand on the properties of ruthenium complexes, about the role of chloride and *β*-diketonate and about the interaction of these complexes with *N*-acetylcysteine and glutathione. Therefore, we have decided to study interactions of the selected ruthenium complexes **1**, **2**, and **3** with *N*-acetylcysteine, glutathione and also with the thiol-containing amino acid cysteine. We have investigated the speciation and binding properties of ruthenium complexes in water and in the presence of additives by electrospray ionisation mass spectrometry (ESI-MS). ESI-MS proved to be a powerful approach to investigate organoruthenium compounds and their interaction with amino acids and larger biomolecules.^12^

## Results and discussion

### ESI mass spectra of aqueous solutions

**Fig. 1:**
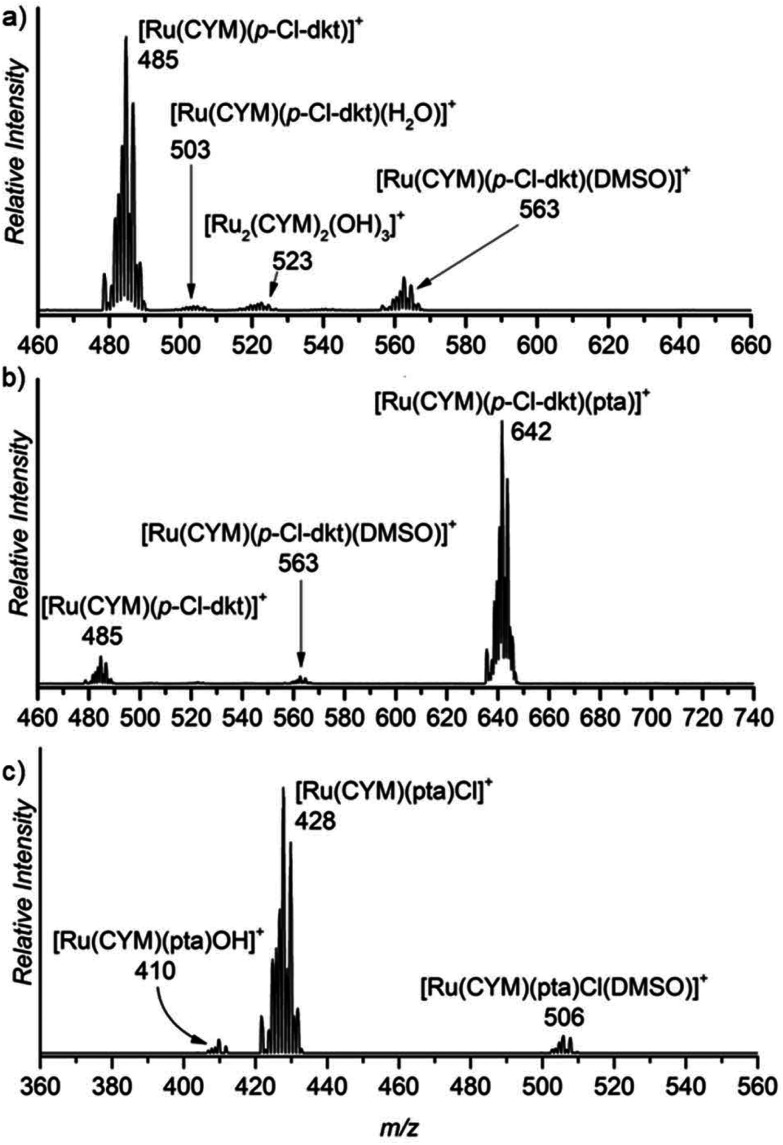
Sections of the ESI mass spectra obtained by spraying 0.2 mM aqueous solution of (a) [Ru(CYM)(*p*-Cl-dkt)]Cl, (b) [Ru(CYM)(pta)(*p*-Cl-dkt)]PF_6_, and (c) RAPTA-C.

First, we studied the speciation of the ruthenium(ii) complexes in water by ESI-MS. The complexes were initially dissolved in a small amount of DMSO, and the solutions were then diluted in water (the exact compositions can be found in Experimental details). The dominant signal in the spectrum of the aqueous solution of [Ru(CYM)(*p*-Cl-dkt)Cl] (**1**) belongs to the ion [Ru(CYM)(*p*-Cl-dkt)]^+^ (*m*/*z* 485, Fig. 1a). A solvent molecule, either H_2_O or DMSO, can occupy the coordination site vacated by the chlorido ligand. We can also observe a small signal of [Ru_2_(CYM)_2_(OH)_3_]^+^ (*m*/*z* 523) that reflects the degradation of complex **1** by ligand-exchange reactions.

The dominant peak in the ESI mass spectrum of **2** is the parent cation [Ru(CYM)(pta)(*p*-Cl-dkt]^+^ (*m*/*z* 642, Fig. 1b). This complex is fully coordinatively saturated; accordingly, we observe no adducts with solvent molecules. Instead, we observe partial fragmentation of the parent complex by loss of the pta ligand. The fragment complex [Ru(CYM)(*p*-Cl-dkt)]^+^ (*m*/*z* 485) has a free coordination site, as discussed above, and therefore associates with either H_2_O or DMSO. The elimination of the pta ligand from the parent cation is most likely induced during the electrospray ionisation process. Thus, we probed the collision-induced dissociation (CID) of the [Ru(CYM)(pta)(*p*-Cl-dkt)]^+^ cation. Indeed, pta elimination is the exclusive fragmentation channel, and the energy-resolved CID experiments provided the appearance energy for the pta elimination, 1.7 ± 0.1 eV. Previous theoretical study reported that anticancer activity of the complexes bearing the pta ligand depended on the Ru–P distance.^14^ The longer the distance, the smaller the anticancer activity. Hence, the bond energy (or ease of the Ru–P bond dissociation) also probably correlates with the biological activity of these complexes.

Electrospray ionisation of an aqueous solution of RAPTA-C (**3**) predominantly leads to the detection of [Ru(CYM)(pta)Cl]^+^ ions (*m*/*z* 428, Fig. 1c). This complex has a free coordination site and therefore a solvent molecule can be added to the complex. Surprisingly, we can only observe DMSO, and not H_2_O, addition. We can also observe a small amount of solvolysis of the chlorido ligand by [Ru(CYM)(pta)OH]^+^ (*m*/*z* 410) detection.

The comparison of ESI mass spectra in Fig. 1 shows no fundamental difference in the speciation of complexes **1–3** in aqueous solution. To the contrary, all complexes are predominantly detected in the expected form of the cation, and there are only minor signs of degradation.

### Experiments in the presence of *N*-acetylcysteine (NAC), glutathione (GSH), cysteine (Cys) and other amino acids

*N*-Acetylcysteine has been used in biological tests of radical oxygen species scavenging. However, its effect was observed after a time delay of several hours.^2^ Hence, the effect of NAC could also be associated with its interaction with ruthenium complexes. Accordingly, we have investigated the effect of NAC addition to the aqueous solutions of **1–3** on the speciation of ruthenium complexes by ESI-MS.

**Fig. 2:**
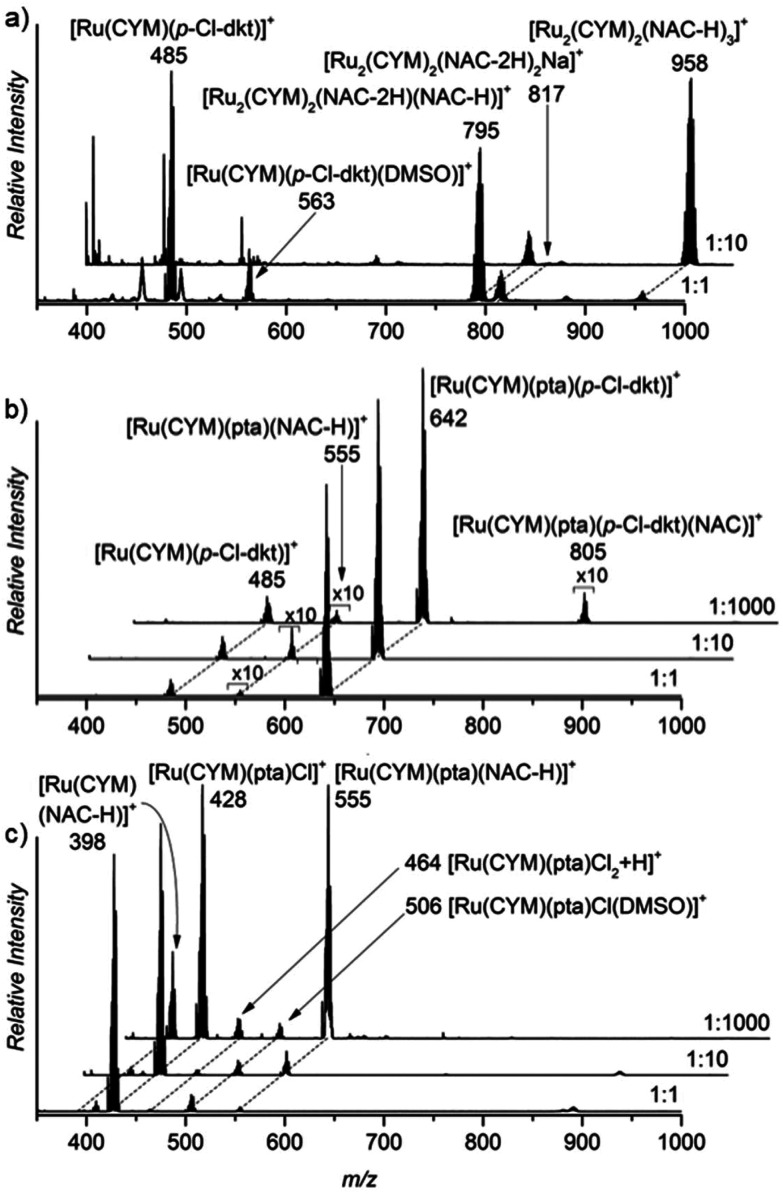
ESI mass spectra of a 0.2 mM aqueous solution of (a) [Ru(CYM)(*p*-Cl-dkt)]Cl (b) [Ru(CYM)(pta)(*p*-Cl-dkt)]PF_6_ and (c) RAPTA-C with NAC. The spectra were acquired immediately after preparing a solution with all components. The molar ratio of the ruthenium(ii) complex to NAC was 1 : 1, 1 : 10 and 1 : 1000 as denoted in each ESI mass spectrum (right bottom).

The ESI mass spectrum of the solution of the first complex, [Ru(CYM)(*p*-Cl-dkt)Cl], shows substantial (*p*-Cl-dkt) ligand exchange with (NAC-H). At an excess of NAC, complete ligand exchange was observed (*cf.* spectra in Fig. 2a measured in molar ratios of [Ru(CYM)(*p*-Cl-dkt)Cl] to NAC being 1 : 1 and 1 : 10). The ruthenium complexes are detected as bimetallic, each ruthenium atom bears the *para*-cymene ligand and the ruthenium centres are bound *via* the deprotonated NAC molecules (Fig. 2a). In comparison, this dramatic change is not observed in the spectra of solutions of the pta-bearing complexes **2** and **3** (Fig. 2b and c). These complexes retain the pta ligand, even in the presence of large excess of NAC (1000 fold excess), and mostly remain as monometallic complexes. The reaction with NAC starts with its coordination to the ruthenium atoms, which more easily occurs in **3** (RAPTA-C) because this complex has one labile coordination site (the site is either empty or filled with a solvent molecule, see above), and the product ion is [Ru(CYM)(pta)(NAC-H)]^+^. Complex **2** reacts with NAC sluggishly, as evidenced by the small signal of the [Ru(CYM)(pta)(*p*-Cl-dkt)(NAC)]^+^ complex. This complex also finally yields [Ru(CYM)(pta)(NAC-H)]^+^, as observed in the spectrum of **2**.

**Fig. 3:**
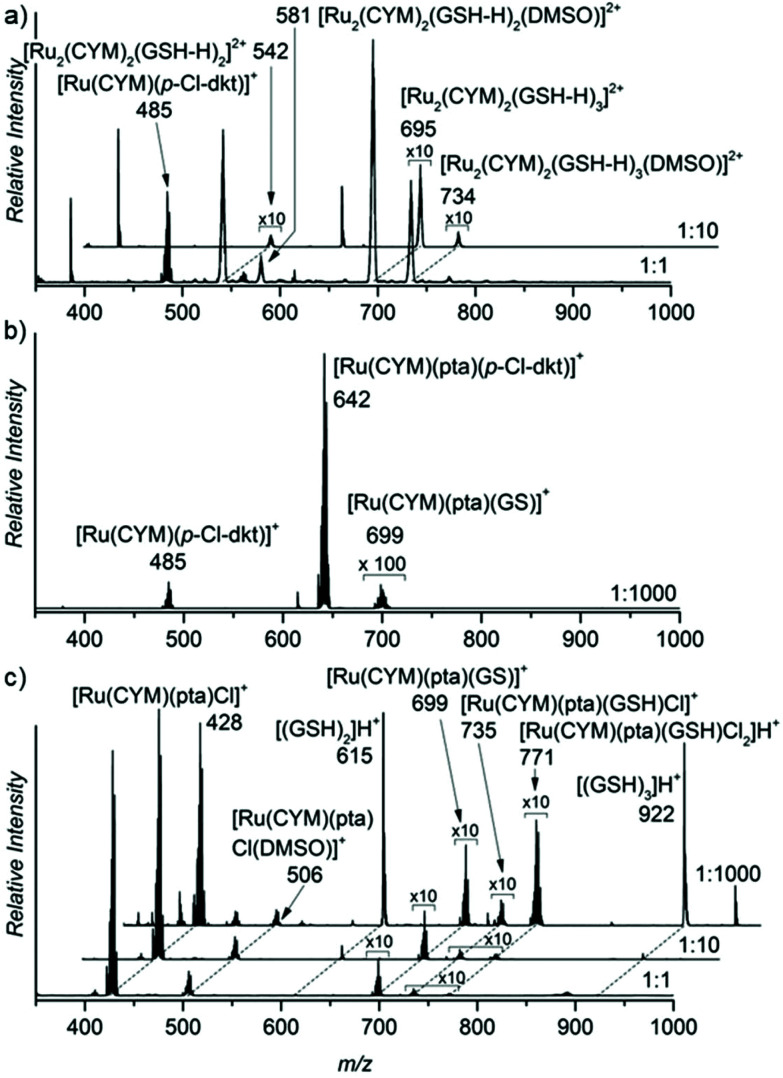
ESI mass spectra of a 0.2 mM aqueous solution of (a) [Ru(CYM)(*p*-Cl-dkt)]Cl, (b) [Ru(CYM)(pta)(*p*-Cl-dkt)]PF_6_ and (c) RAPTA-C with GSH. The spectra were acquired immediately after preparing a solution with all components. The molar ratio of the ruthenium(ii) complex to GSH was 1 : 1, 1 : 10 and 1 : 1000 as denoted in each ESI mass spectrum (right bottom).

Furthermore, we assessed the effect of glutathione addition to the aqueous solutions of the ruthenium complexes (Fig. 3). Glutathione is a tripeptide of glycine, cysteine and gamma-bound glutamic acid. The results were very similar to the findings with NAC. Again, complex **1**, without pta ligand, formed biruthenium clusters bound *via* deprotonated GSH molecules. Complexes **2** and **3** maintained their monometallic stoichiometry, and part of the complexes exchanged their anion by deprotonated GSH. Hence, we observed [Ru(CYM)(pta)(GS)]^+^ ions in the ESI mass spectra of both **2** and **3**.^15^ Anion exchange in complex **2** is very limited because the *β*-diketonate present in the original complex is bidentately coordinated to ruthenium.

*N*-Acetyl cysteine and glutathione contain a thiol group likely responsible for binding to ruthenium atoms. Therefore, we repeated the experiments with bare cysteine, with similar results. Complex **1** formed cysteine-bound biruthenium clusters, whereas **2** and **3** partly underwent anion-exchange with deprotonated cysteine (see Fig. S1 in the ESI†).

**Fig. 4:**
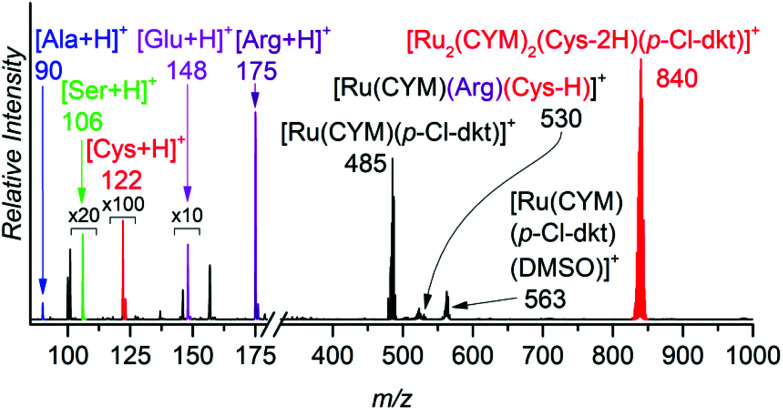
ESI mass spectrum of a 0.2 mM aqueous solution of [Ru(CYM)(*p*-Cl-dkt)]Cl with equimolar concentrations of Cys, Ala, Ser, Glu and Arg. The spectra were acquired immediately after preparing a solution with all components.

To show that ruthenium complexes preferentially interact with thiols, we controlled their speciation in aqueous solution of these complexes with equimolar concentrations of cysteine, alanine, serine, glutamic acid and arginine in one experiment (Fig. 4) and with equimolar concentration of cysteine, histidine, methionine, and aspartic acid in another experiment (Fig. S2†). The results clearly show that cysteine binds much more strongly to the ruthenium than to all other amino acids tested (Fig. 4 and Fig. S2 in the ESI†). We also studied the interaction of ruthenium complexes with other amino acids individually. The results clearly show that the ligand exchange with alanine, serine and glutamic acid is much slower and that the binding energies of these amino acids (amine) are lower than (or similar to, in the case of glutamic acid) that of *β*-diketonate. Ligand exchange with arginine is faster than with alanine, serine and glutamic acid but still slower than with cysteine, leading to different monoruthenium complexes instead of dimerization occuring with cysteine. The binding energy of arginine is higher than that of *β*-diketonate. These results can be found in ESI (Fig. S3–S8†).

In summary, this set of experiments revealed several key findings. (1) All complexes undergo an exchange of anionic ligands with thiol-group-containing molecules. Chloride is exchanged faster than *β*-diketonate. (2) Ruthenium complexes form bimetallic clusters with bridging cysteine-like molecules. (3) The pta ligand remains coordinated to ruthenium, even in the presence of thiol-containing molecules, thereby preventing the formation of larger ruthenium clusters.

### Going deeper: collision-induced dissociation experiments

After establishing the importance of the pta units for the stability of the ruthenium complexes, we compared the binding energies of different ligands in ruthenium complexes in energy-resolved collision-induced dissociation (CID) experiments.^16^

**Fig. 5:**
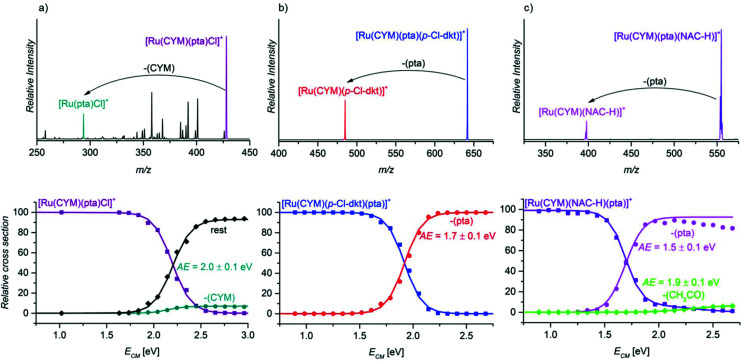
(a) The CID spectra of mass-selected ions (a) [Ru(CYM)(pta)Cl]^+^ (*E*_coll_ 2.2 eV at the center of mass frame, c.m.), (b) [Ru(CYM)(pta)(*p*-Cl-dkt)]^+^ (*E*_coll_ 1.8 eV, c.m.) and (c) [Ru(CYM)(pta)(NAC-H)]^+^ (*E*_coll_ 1.5 eV, c.m.), and their breakdown curves as function of collision energies.

First, we compared the binding energies of the pta ligand in different ruthenium complexes: [Ru(CYM)(pta)(anion)]^+^ (anion = Cl, *p*-Cl-dkt, NAC-H, GS, Cys-H), and [Ru(CYM)(pta)(*p*-Cl-dkt)(NAC)]^+^. The collisional activation of the complex [Ru(CYM)(pta)(Cl)]^+^ (Fig. 5a) induces many different fragmentation pathways, mostly involving eliminations of parts of the ligands. We can also observe HCl and *para*-cymene elimination. All fragmentations have similar appearance energies of approximately 2.0 eV (we assessed all fragmentation channels together; only the CYM elimination is plotted separately). In comparison, fragmentation of all other complexes [Ru(CYM)(pta)(anion)]^+^ (*i.e.* anion = *p*-Cl-dkt, NAC-H, GS, Cys-H) leads to exclusive elimination of the pta ligand (Fig. 5b, c and Fig. S10 and S11 in the ESI†). The estimated pta bonding energy is 1.7 ± 0.1 eV in [Ru(CYM)(pta)(*p*-Cl-dkt)]^+^ (Fig. 5b), 1.5 ± 0.1 in [Ru(CYM)(pta)(NAC-H)]^+^ (Fig. 5c), and identically 1.3 ± 0.1 eV for [Ru(CYM)(pta)(GS)]^+^ (Fig. S10 in the ESI†) and [Ru(CYM)(pta)(Cys-H)]^+^ (Fig. S11 in the ESI†).

This series of measurements of pta binding energies reveals that the deprotonated cysteine-based ligands stabilise the fragment ruthenium complex slightly better than the *β*-diketonato ligand. Hence, the formation of [Ru(CYM)(NAC-H)]^+^/[Ru(CYM)(GS)]^+^/[Ru(CYM)(Cys-H)]^+^ demands less energy than that of [Ru(CYM)(*p*-Cl-dkt)]^+^. Accordingly, the anions (NAC-H)^−^, GS^−^, and (Cys-H)^−^ are coordinated to ruthenium as bidentate ligands. The fragment complexes can further dissociate by elimination of a part of the deprotonated ligand with a higher energy onset (see Fig. 5c and Fig. S10 in the ESI†). These subsequent fragmentations were confirmed by MS/MS/MS (Fig. S12 and S13 in the ESI†). In comparison, the formation of [Ru(CYM)(Cl)]^+^ is virtually suppressed because it is much more energy-demanding than other fragmentation channels.

The larger stabilisation of the ruthenium complexes by deprotonated NAC/GS/Cys than by *β*-diketonate and chloride is in line with the speciation of complex **1**, [Ru(CYM)(*p*-Cl-dkt)Cl], in the presence of NAC/GS/Cys. The complete transformation of complex **1** into bimetallic complexes of the [Ru_2_(CYM)_2_(NAC-H)_3_]^+^ type also indicates that deprotonated amino acids/tripeptide with a thiol group are more strongly bound ligands than the *β*-diketonate.

**Fig. 6:**
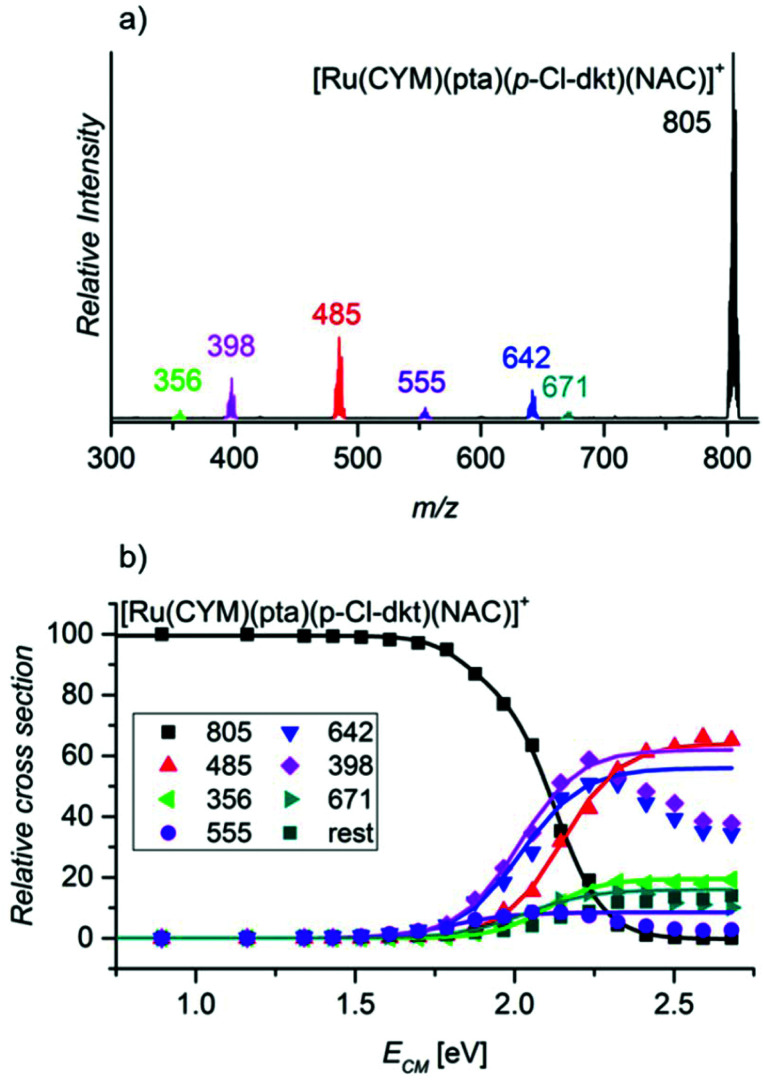
(a) The CID spectrum of mass-selected [Ru(CYM)(pta)(*p*-Cl-dkt)(NAC)]^+^ (*E*_coll_ 2.1 eV, c.m.) and (b) breakdown curves. Breakdown curves of the fragments with *m*/*z* 671, 642, 555, 398 and 356 were enlarged five times.

Finally, we probed the fragmentation of [Ru(CYM)(pta)(*p*-Cl-dkt)(NAC)]^+^ (Fig. 6). Most likely, this complex is an intermediate of the association–dissociation ligand exchange reaction. Hence, we should observe either NAC or neutral ketoenol (protonated *β*-diketonate, *p*-Cl-dkt·H) ligand elimination. Surprisingly, the fragmentation of [Ru(CYM)(pta)(*p*-Cl-dkt)(NAC)]^+^ is much more complex and many possible fragmentation channels occur with similar appearance energies (Table 2).

**Table 2:** Observed fragments and appearance energies determined by energy-resolved CID of the mass-selected ion [Ru(CYM)(pta)(*p*-Cl-dkt)(NAC)]^+^

Fragment	*m*/*z*	Eliminated fragment(s)	AE/eV
[Ru(pta)(*p*-Cl-dkt)(NAC)]^+^	671	CYM	1.8 ± 0.1
[Ru(CYM)(pta)(*p*-Cl-dkt)]^+^	642	NAC	1.8 ± 0.1
[Ru(CYM)(pta)(NAC-H)]^+^	555	(*p*-Cl-dkt)H	1.6 ± 0.1
[Ru(CYM)(*p*-Cl-dkt)]^+^	485	NAC + pta	2.0 ± 0.1
[Ru(CYM)(NAC-H)]^+^	398	*p*-Cl-dkt·H + pta	1.8 ± 0.1
[Ru(CYM)((NAC-H)-CH_2_CO)]^+^	356	*p*-Cl-dkt·H + pta + CH_2_CO	2.0 ± 0.1

The least energy-demanding pathway of [Ru(CYM)(pta)(*p*-Cl-dkt)(NAC)]^+^ fragmentation leads to the expected *p*-Cl-dkt·H elimination with an appearance energy of 1.6 ± 0.1 eV. The appearance energy of NAC elimination is 1.8 ± 0.1 eV. Unexpectedly, *para*-cymene elimination has the same appearance energy (1.8 ± 0.1 eV), whereas the pta ligand is eliminated after the initial loss of *p*-Cl-dkt·H or NAC with an energy difference of only 0.2 eV. The fragmentation pattern shows that *β*-diketonate and NAC have a smaller binding energy to ruthenium than pta in [Ru(CYM)(pta)(*p*-Cl-dkt)(NAC)]^+^. Previous CID experiments (see Fig. 5) have indicated that pta elimination is the exclusive fragmentation channel in complexes with one ligand. Most likely, [Ru(CYM)(pta)(*p*-Cl-dkt)(NAC)]^+^ differs from [Ru(CYM)(pta)(anion)]^+^ (anion = *β*-diketonate or NAC) because *β*-diketonate and NAC can only bind as monodentate ligands in the [Ru(CYM)(pta)(*p*-Cl-dkt)(NAC)]^+^ complex, whereas they are bidentate in [Ru(CYM)(pta)(anion)]^+^.

Thus, this experiment shows how the pta ligand protects ruthenium complexes from degradation. The coordinatively saturated complex [Ru(CYM)(pta)(*p*-Cl-dkt)]^+^ can accept the additional ligand only by changing the coordination mode of *β*-diketonate (*p*-Cl-dkt) from bidentate to monodentate. Upon this change, both *p*-Cl-dkt and NAC are more weakly bound to ruthenium than the pta ligand, albeit with a rather high binding energy. Hence, we can only observe the exchange of the anionic ligand, whereas the pta ligand remains coordinated to ruthenium and prevents the formation of biruthenium clusters because pta is a bulky ligand.

### Going deeper: infrared photodissociation spectroscopy

**Fig. 7:**
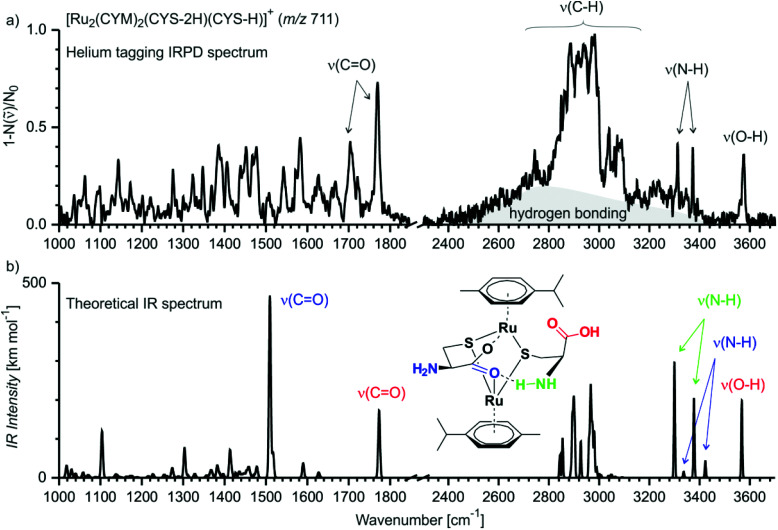
(a) Helium-tagging IRPD spectrum of mass-selected ion [Ru_2_(CYM)_2_(Cys-2H)(Cys-H)]^+^ (*m*/*z* 711). (b) Theoretical IR spectrum (B3LYP-D3/6-31G**:SDD(Ru)) of one of the possible isomers of [Ru_2_(CYM)_2_(Cys-2H)(Cys-H)]^+^ (IR spectra of other isomers are shown in Fig. S14,† the *xyz* coordinates for all reported structures are in the ESI† and can be visualised).

Above, we showed that thiol-containing amino acids/tripeptide strongly bind to ruthenium forming biruthenium complexes. However, the structure of these biruthenium complexes is unknown. In the gas phase, the structure of ionic complexes can be more directly deduced from their infrared photodissociation (IRPD) spectra.^17^ Thus, we measured the IRPD spectrum of [Ru_2_(CYM)_2_(Cys-2H)(Cys-H)]^+^ (Fig. 7). We have chosen this complex because it was the simplest bimetallic complex for explorative theoretical calculations of IR spectra.

The IRPD spectrum of [Ru_2_(CYM)_2_(Cys-2H)(Cys-H)]^+^ shows one free O–H stretching vibration at 3575 cm^−1^ and two N–H stretching vibrations at 3373 cm^−1^ and 3312 cm^−1^, with no S–H vibration around 2500 cm^−1^.^18^ We can also observe a broad, intense background in the range of C–H vibrations (2500–3300 cm^−1^; highlighted by grey in Fig. 7). This spectral feature is most likely a progression of stretching vibrations of (N–H) bonds involved in hydrogen bonding. In the fingerprint region, we observe two C=O vibrations at 1770 cm^−1^ and 1705 cm^−1^. All this spectral information is consistent with a structure of the complex in which ruthenium atoms are bound *via* two thiolate and one or both carboxylate groups, and the carboxylate group is most likely bridging the ruthenium atoms.

We have further explored possible structures of the [Ru_2_(CYM)_2_(Cys-2H)(Cys-H)]^+^ complex by DFT calculations. The most stable structures contain a motif of the ruthenium atoms bound by two sulfur bridges and by a carboxylate bridge (Fig. 7b, the drawing indicates the optimised structure, the actual structure can be visualised using *xyz* coordinates reported in the ESI†). The calculations nicely reproduce the detected OH and NH bands. Moreover, the free carbonyl group is identified in the same spectral position as that detected experimentally. The carbonyl bond involved in bridging the ruthenium atoms is redshifted to 1500 cm^−1^.^19^ The theoretical spectrum coherently explains the main peaks of the IRPD spectrum, but not all of them because this complex is large and flexible. Thus, we are most likely probing a mixture of different isomers/conformers of [Ru_2_(CYM)_2_(Cys-2H)(Cys-H)]^+^ when performing these IRPD analysis. Particularly hydrogen bridges within the complex can change, which leads to different shifts in X–H (X = O, N) and C=O vibrations. For other localised structures in our exploratory survey, see ESI (Fig. S14†).

We have also considered possible formation of cystine with S–S bond and its presence in the detected ruthenium complexes (Fig. S15†). Oxidation of the cysteine ligands would have to be associated with reduction of the ruthenium atoms. We have localised two possible isomers of such complexes. Relative energy of these complexes is much higher (∼300 kJ mol^−1^) than those of the initially considered ruthenium(ii) complexes. The sulfur atoms are not available for coordination to the ruthenium atoms, therefore both carboxyl functions and one of the amino groups are involved in coordination to and bridging of the ruthenium centers. Consequently, the IR signatures of the carboxyl and the amino groups do not agree with the experimental IRPD spectrum. Hence, we can exclude that we detected ruthenium complexes with an oxidised cystine ligand.

Finally, we also measured the IRPD spectrum of [Ru_2_(CYM)_2_(NAC-H)_3_]^+^ (Fig. S16 in ESI†). The spectrum shows no S–H vibration, thus also confirming that the ruthenium atoms are bound *via* thiolate bridges. The spectrum has broader bands than the spectrum of [Ru_2_(CYM)_2_(Cys-2H)(Cys-H)]^+^. Moreover, the ruthenium atoms are bridged by three ligands. Therefore, we most likely probed an even more complex mixture of isomers/conformers than in the case of [Ru_2_(CYM)_2_(Cys-2H)(Cys-H)]^+^.

### Implications for the biological activity of ruthenium complexes

(1) Our results show that the pta ligand protects the ruthenium complexes from ligand exchange with water and from the formation of biruthenium clusters.

(2) The ruthenium complexes readily interact with cysteine-derived molecules at the sulfur atom. Ruthenium complexes without the pta ligand form bimetallic clusters bound *via* the deprotonated thiol groups, whereas pta-bearing ruthenium complexes remain monometallic and attach only one cysteine-derived molecule.

(3) The *β*-diketonato ligand slows down reactions with cysteine-derived molecules. Hence, although we observe immediate exchange of chlorido ligands by deprotonated cysteines, *β*-diketonato ligand exchange is slow.

Based on the above, assuming that the biological activity of ruthenium complexes, regarding the formation of reactive oxygen species, is associated with their clustering, our results explain why the biological activity of pta-bearing ruthenium complexes is not based on ROS production. Both complexes with pta ligands do not form bimetallic species, whereas the other does so.

In addition, assuming that the biological activity of monoruthenium complexes is associated with interactions with peptides, our results show that the ruthenium complexes would bind to the cysteine residues. The addition of other cysteine-derived molecules, such as *N*-acetylcysteine, would lead to a competitive binding to the ruthenium complexes, thereby decreasing their biological activity. Similarly, if we decrease the levels of molecules that can competitively bind to the ruthenium complexes, such as glutathione, we will increase the biological activity of these complexes. The effect would be stronger on [Ru(CYM)(pta)(Cl)]^+^ than on [Ru(CYM)(pta)(*p*-Cl-dkt)]^+^ because the former interacts more quickly with thiols and is thus less selective than the latter. Although these experiments were performed in solution, without the complex biological matrix, these inferences based on our results perfectly corroborate previous biological findings.

## Conclusions

We conducted a comparative study of ruthenium complexes with different ligands ([Ru(CYM)(*p*-Cl-dkt)(Cl)], [Ru(CYM)(pta)(*p*-Cl-dkt)]PF_6_, [Ru(CYM)(pta)Cl_2_]). These complexes have been previously tested for their anti-cancer activities and shown to be biologically active, albeit with different mechanisms. We also showed that these complexes have different stabilities and thus speciations in aqueous solution by mass spectrometry. The pta ligand has a significant stabilisation effect and stabilises the monoruthenium complexes against formation of larger clusters. Conversely, the [Ru(CYM)(*p*-Cl-dkt)(Cl)] complex undergoes solvolysis and forms bimetallic [Ru_2_(CYM)_2_(OH)_3_]^+^ complexes. All ruthenium complexes reacted with a thiol group-bearing amino acid (cysteine), amino acid derivative (*N*-acetylcysteine) and tripeptide (glutathione). The reactions with other amino acids (alanine, methionine, serine, glutamic acid, aspartic acid, arginine, histidine) are much slower. The [Ru(CYM)(*p*-Cl-dkt)(Cl)] complex reacts faster with cysteine-like molecules than both pta-bearing complexes analysed. In addition, the [Ru(CYM)(pta)(*p*-Cl-dkt)]^+^ complex reacts with cysteine and cysteine-derived molecules more slowly than the [Ru(CYM)(pta)(Cl)]^+^ complex because the bidentate *β*-diketonato ligand must change its coordination mode upon thiol addition. Lastly, we studied the binding mode of cysteine and cysteine-like molecules by infrared photodissociation spectroscopy, and the results showed that these molecules are deprotonated at the sulfur atom and that sulfur bridges the ruthenium atoms in these bimetallic complexes.

## Experimental

The synthesis of the ruthenium(ii) complexes has been previously reported.^2,20^ Stock solutions of ruthenium(ii) complexes were prepared in 3% DMSO/water. The different amino acids and tripeptide were prepared only in water. Stock solutions were diluted in water, mixed in the same vial and immediately subjected to mass spectrometry or ion spectroscopy. The concentration of ruthenium(ii) complexes was kept constant, and the solution of amino acids and tripeptide was added at different ratios. The ratio of ruthenium(ii) complexes to amino acids/tripeptide is indicated in each ESI mass spectrum. For the initial experiments, [Ru(CYM)(pta)(*p*-Cl-dkt)]PF_6_ was prepared in 3% DMSO/water. Conversely, for the experiments with NAC, GSH and Cys, [Ru(CYM)(pta)(*p*-Cl-dkt)]PF_6_ was prepared only in water. Details on the preparation of the stock solutions are included in ESI.†

ESI mass spectra were acquired with a linear ion trap instrument LTQ or a triple quadrupole mass spectrometer TSQ 7000 equipped with an electrospray ionisation source. For TSQ 7000, the following conditions were used: 4.5 kV electrospray voltage, 250 °C capillary temperature, 30 psi sheath gas pressure, 6 μl min^−1^ flow rate, −10 V capillary voltage and 70 V lens voltage. For LTQ, the following conditions were used: 5.5 kV electrospray voltage, 250 °C capillary temperature, 50 psi sheath gas pressure, 6 μl min^−1^ flow rate, 0 V capillary voltage and 70 V lens voltage. Energy-resolved CID experiments were performed using a Finnigan LCQ Deca mass spectrometer equipped with an electrospray ionisation source. The excitation period was 30 ms, and the trapping parameter was *q*_*z*_ = 0.25 for mass-selected ions. The Schröder's calibration method was used to calibrate the collision energy and to determine the appearance energies.^21^ The calibration was performed by correlating the experimental appearance energies of a set of substituted benzylpyridiunium ions [RBnPy]^+^ with the values determined by Carpenter *et al.* (Fig. S9 in the ESI†).^22^ The values of the appearance energies are given with the experimental uncertainties of three different measurements.

IRPD spectra were acquired using the ISORI^23^ (Ion Spectroscopy Of Reaction Intermediates) instrument based on the TSQ 7000 platform. Ionisation and mass selection were performed similarly to classical experiments with TSQ 7000. Mass-selected ions are trapped in an ion trap and cooled to 3 K. The trapped ions form loosely bound complexes with the helium buffer gas. Helium complexes are then used to determine the absorption of IR photons. The IR photon absorption leads to helium detachment, thereby enabling its detection as the change in *m*/*z* ratio by mass spectrometry. The number of helium complexes with (*N*_*i*_) and without (*N*_*i*0_) laser irradiation is counted using a Daly-type detector. The IRPD spectra are constructed as (1 − *N*_*i*_/*N*_*i*0_). More details can also be found in ref. 14, 20 and 24.

The theoretical results were obtained using the density functional theory at the B3LYP^25–28^ level with D3 semiempirical corrections of dispersion interactions.^29^ The basis set was 6-31G** for all atoms, except ruthenium, for which we used the SDD pseudopotential basis set. The randomly suggested structures were fully optimised, and the minima were then characterised by frequency calculations. The theoretical IR spectra were scaled by 0.96 in the fingerprint range and by 0.95 above 2500 cm^−1^.

## Conflicts of interest

There are no conflicts to declare.

## Supplementary Material

DT-048-C8DT04350G-s001
