# Supplementary material for: Anti-cancer organoruthenium(ii) complexes and their interactions with cysteine and its analogues. A mass-spectrometric study
Source: Dalton Trans. 2019 Jan 28;48(8):2626–34. doi: 10.1039/c8dt04350g (PMC8609305; doi:10.1039/c8dt04350g)
Supplement: DT-048-C8DT04350G-s001 [file DT-048-C8DT04350G-s001.pdf]

Electronic Supplementary Material (ESI) for Dalton Transactions.
This journal is © The Royal Society of Chemistry 2019

Supporting Information

Anti-cancer organoruthenium(II) complexes and their interactions with cysteine and its analogues. A mass-spectrometric study.

Anamarija Briš,[a,b] Juraj Jašík,[a] Iztok Turel[c] and Jana Roithová[a,d]*

[a] Department of Organic Chemistry, Faculty of Science, Charles University, Hlavova 2030/8, 128 43 Prague, Czech Republic
[b] Ruđer Bošković Institute, Bijenička 54, 10 000 Zagreb, Croatia
[c] Faculty of Chemistry and Chemical Technology, University of Ljubljana, Večna pot 113, SI-1000 Ljubljana, Slovenia
[d] Institute for Molecules and Materials, Radboud University, Heyendaalseweg 135, 6525 AJ Nijmegen, The Netherlands,
E-mail: jana.roithova@ru.nl

Contents

Preparation of reaction mixtures S2
ESI mass spectra and CID spectra S3
IRPD and theoretical IR spectra S9
Computational results S11

Preparation of reaction mixtures

Stock solutions of ruthenium(II) complexes and different amino acids/tripeptide were prepared and stored for no longer than 2 days.

Table S1. Contents of the stock solutions of ruthenium(II) complexes.

Stock Solution	Reagent	Quantity (mg)	n (µmol)	Solvent	Quantity (µl)
**I**	[Ru(CYM)(*p*-Cl-dkt)]	1	2	3 % DMSO in water	5000
**II**	[Ru(CYM)(pta)(*p*-Cl-dkt)]PF_6	1	1	3 % DMSO in water	3200
**IIa**	[Ru(CYM)(pta)(*p*-Cl-dkt)]PF_6	1	1	Water	3200
**III**	RAPTA-C	1	2	3 % DMSO in water	5400

Stock solutions of NAC, GSH and Cys were prepared in two different concentrations. The higher concentration solutions were used in measurements with 1000 equivalents of amino acid/tripeptide to ruthenium(II) complexes.

Table S2. Contents of the stock solutions of different amino acids/tripeptide.

Stock Solution	Reagent	Quantity (mg)	n	Solvent	Quantity (µl)
**NAC I**	*N*-acetylcysteine	1	6 µmol	Water	2000
**NAC II**	*N*-acetylcysteine	100	0.6 mmol	Water	2000
**GSH I**	Glutathione	1	3 µmol	Water	1000
**GSH II**	Glutathione	100	0.3 mmol	Water	1000
**Cys I**	L-Cysteine	1	8 µmol	Water	2500
**Cys II**	L-Cysteine	100	0.8 mmol	Water	2500
**Ala I**	L-Alanine	1	10 µmol	Water	2500
**Ser I**	L-Serine	1	10 µmol	Water	2500
**Glu I**	L-Glutamic acid	1	7 µmol	Water	2500
**Arg I**	L-Arginine	1	6 µmol	Water	1000
**His I**	L-Histidine	1	6 µmol	Water	1600
**Met I**	L-Methionine	1	7 µmol	Water	1700
**Asp I**	L-Aspartic acid	1	8 µmol	Water	1900

ESI mass spectra and CID spectra

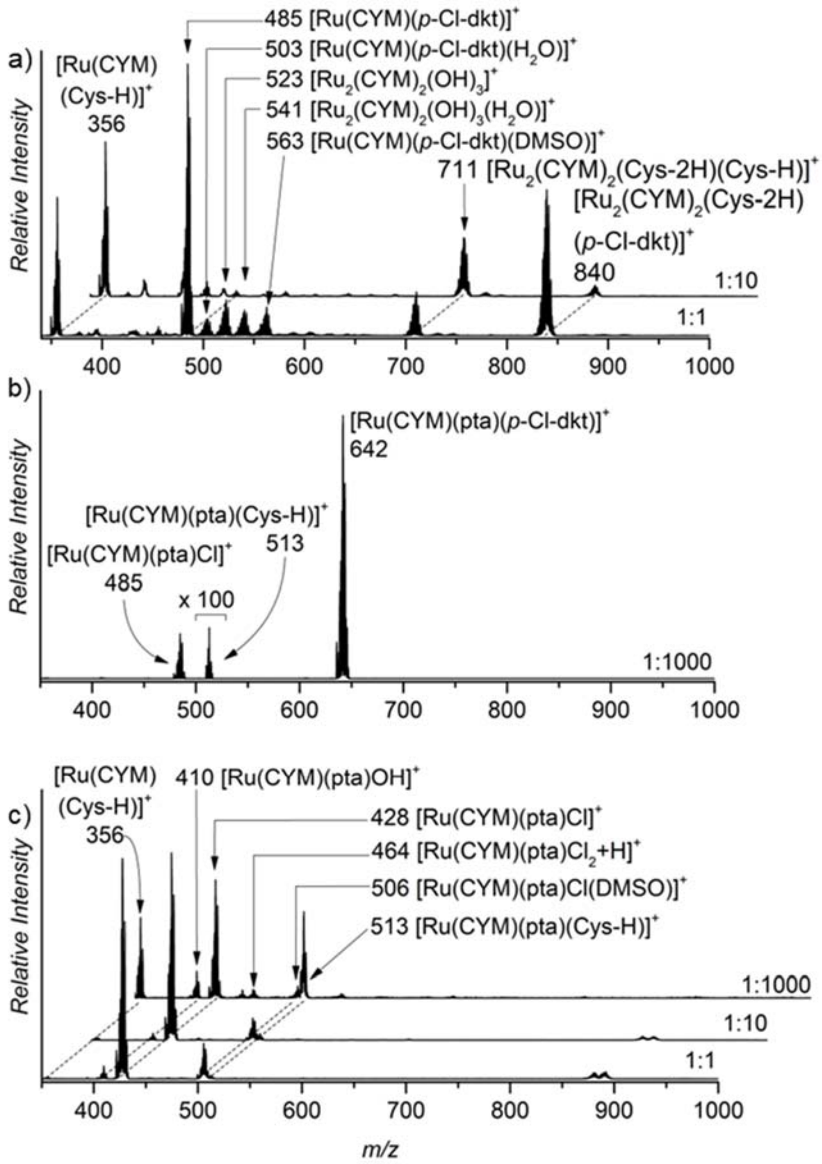

Figure S1. ESI mass spectra of 0.2 mM aqueous solution of a) [Ru(CYM)(*p*-Cl-dkt)]Cl, b) [Ru(CYM)(pta)(*p*-Cl-dkt)]PF_6 and c) RAPTA-C with Cys. The spectra were acquired immediately after preparing a solution with all components. The molar ratio of the ruthenium(II) complex to Cys was 1:1, 1:10 and 1:1000 as denoted in each ESI mass spectrum (right bottom).

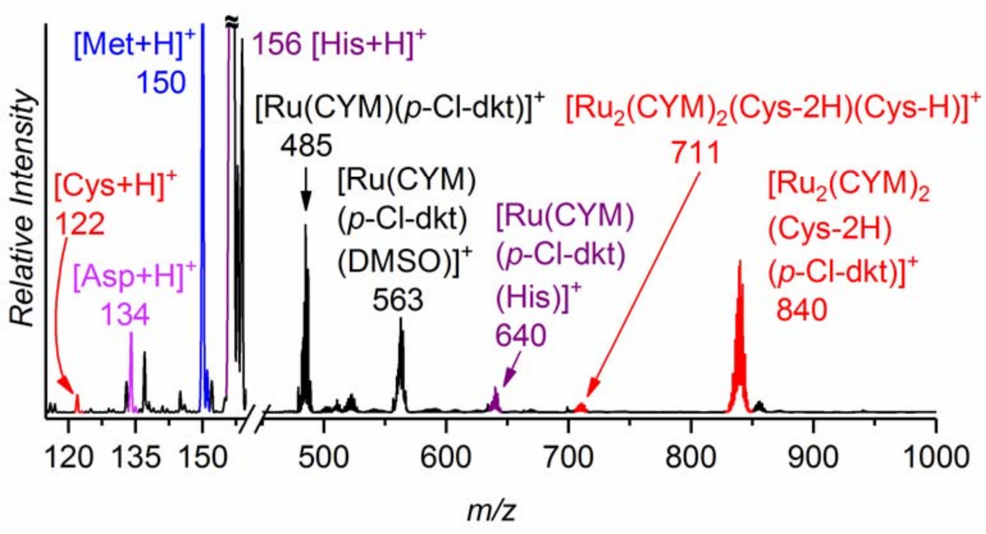

Figure S2. ESI mass spectrum of a 0.2 mM aqueous solution of [Ru(CYM)(*p*-Cl-dkt)]Cl with equimolar concentrations of Cys, Asp, Met, and His. The spectra were acquired immediately after preparing a solution with all components.

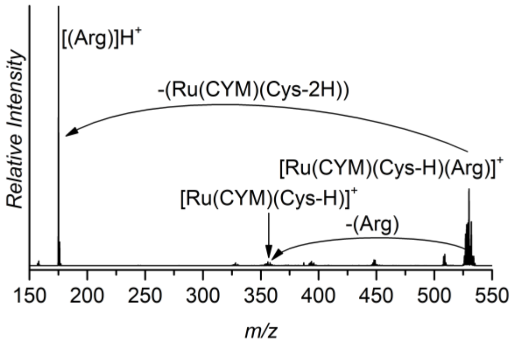

Figure S3. The CID spectrum of mass-selected ion [Ru(CYM)(Cys-H)(Ala)]$^{+}$.

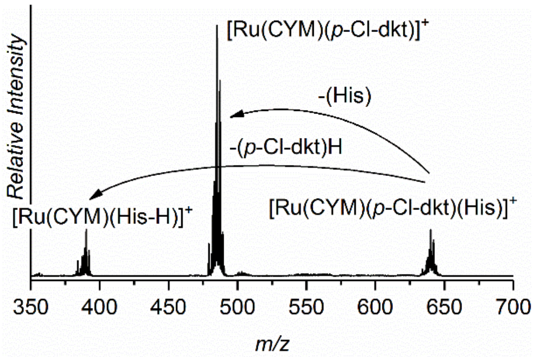

Figure S4. The CID spectrum of mass-selected ion [Ru(CYM)(*p*-Cl-dkt)(His)]$^{+}$.

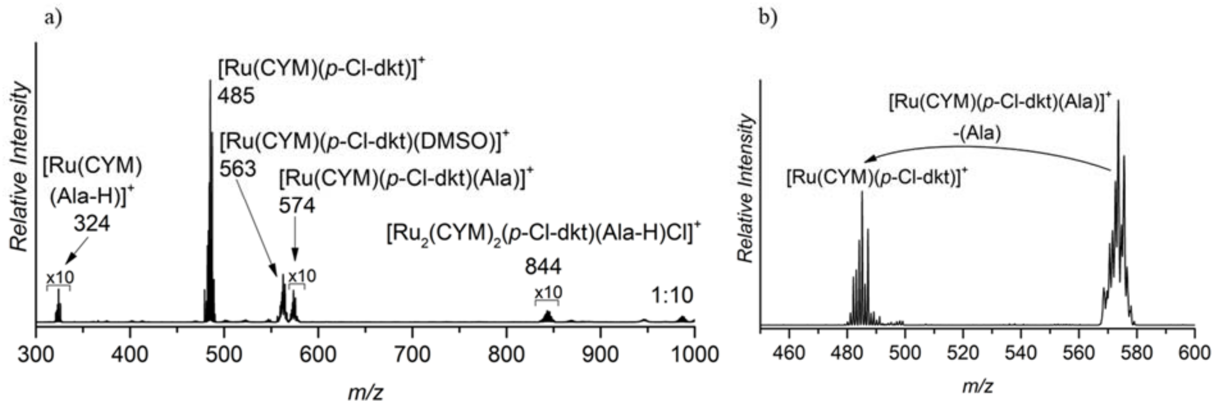

Figure S5. a) ESI mass spectra of 0.2 mM aqueous solution of [Ru(CYM)(*p*-Cl-dkt)]Cl with Ala and b) the CID spectrum of mass-selected ion $[Ru(CYM)(p\text{-}Cl\text{-}dkt)(Ala)]^{+}$. The spectra were acquired immediately after preparing a solution with all components. The [Ru(CYM)(*p*-Cl-dkt)]Cl to Ala ratio was 1 to 10.

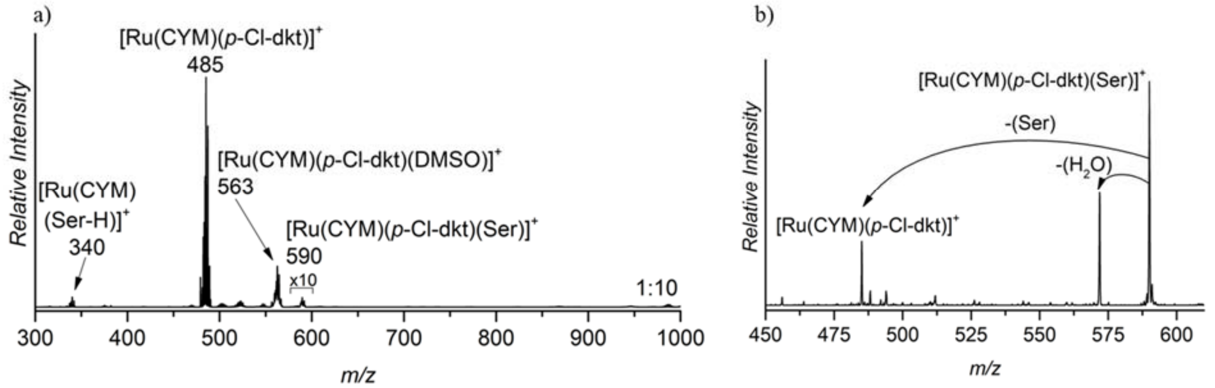

Figure S6. a) ESI mass spectra of 0.2 mM aqueous solution of [Ru(CYM)(*p*-Cl-dkt)]Cl with Ser and b) the CID spectrum of mass-selected ion $[Ru(CYM)(p\text{-}Cl\text{-}dkt)(Ser)]^{+}$. The spectra were acquired immediately after preparing a solution with all components. The [Ru(CYM)(*p*-Cl-dkt)]Cl to Ser ratio was 1 to 10.

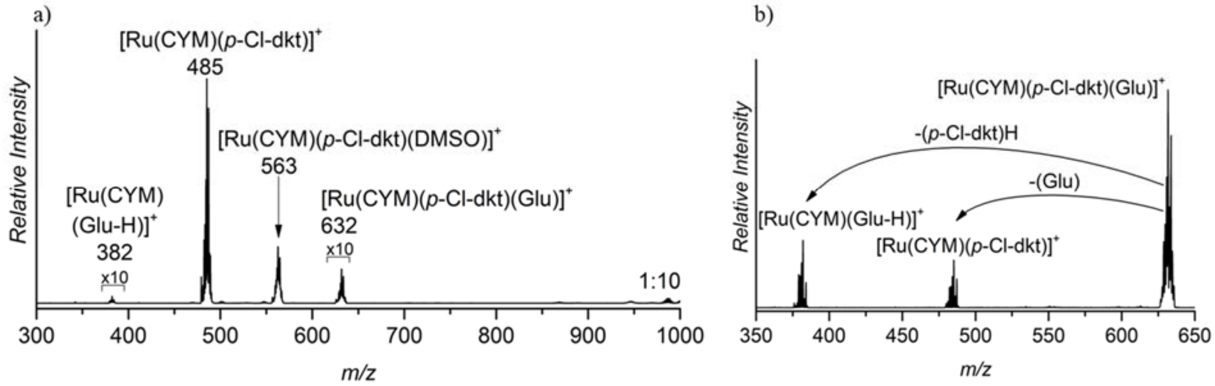

Figure S7. a) ESI mass spectra of 0.2 mM aqueous solution of [Ru(CYM)(*p*-Cl-dkt)]Cl with Glu and b) the CID spectrum of mass-selected ion $[Ru(CYM)(p\text{-}Cl\text{-}dkt)(Glu)]^{+}$. The spectra were acquired immediately after preparing a solution with all components. The [Ru(CYM)(*p*-Cl-dkt)]Cl to Glu ratio was 1 to 10.

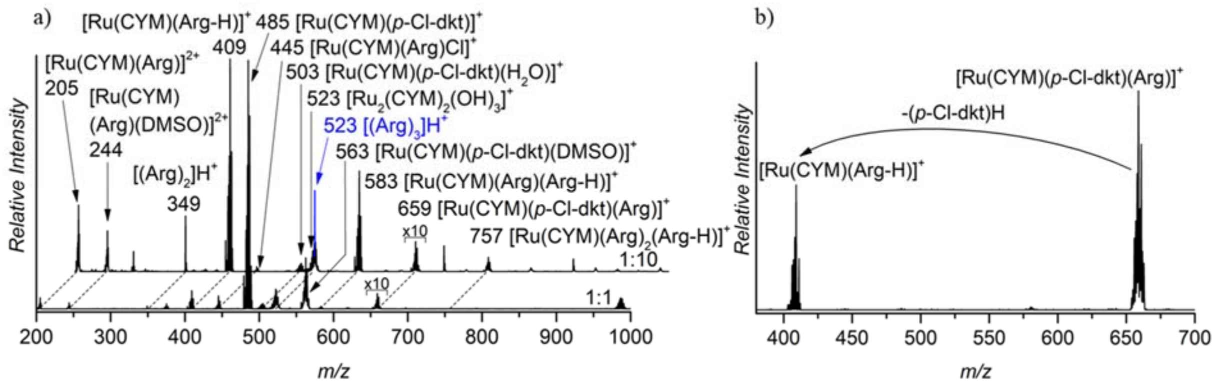

Figure S8. a) ESI mass spectra of 0.2 mM aqueous solution of [Ru(CYM)(*p*-Cl-dkt)]Cl with Arg and b) the CID spectrum of mass-selected ion [Ru(CYM)(*p*-Cl-dkt)(Arg)]$^+$. The spectra were acquired immediately after preparing a solution with all components. The molar ratio of the ruthenium(II) complex to Arg was 1:1 and 1:10 as denoted in each ESI mass spectrum (right bottom).

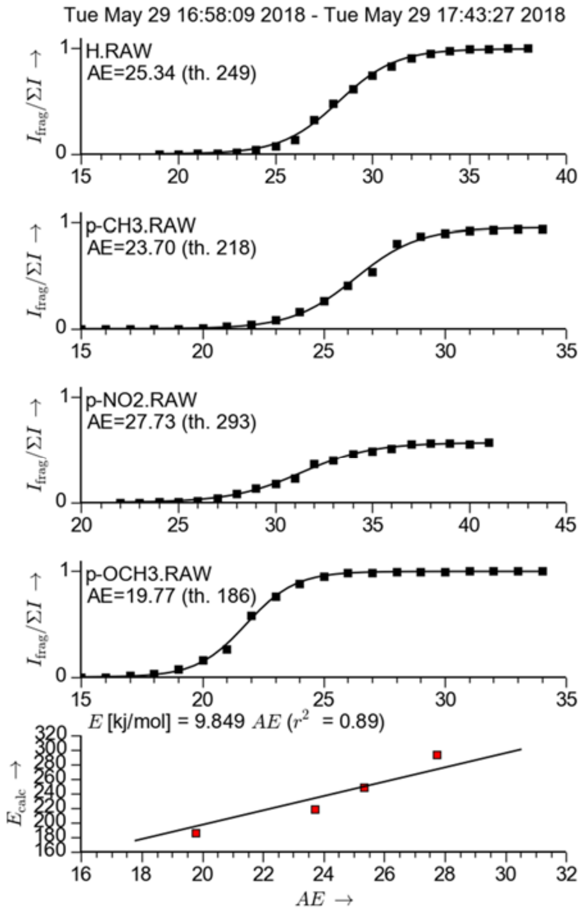

Figure S9. Calibration curves and sigmoidal fits of the relative intensities in dependence to normalised collision energies for positive-ion mode for substituted benzylpyridiunium ions $[RBnPy]^+$. The linear extrapolation of the sigmoidal fit rise gives appearance energy. Used $[RBnPy]^+$ were benzylpyridinium (R=H), *p*-methylbenzylpyridinium (R=*p*-CH3), *p*-methoxybenzylpyridinium (R=*p*-OCH3) and *p*-nitrobenzylpyridinium (R=*p*-NO2).

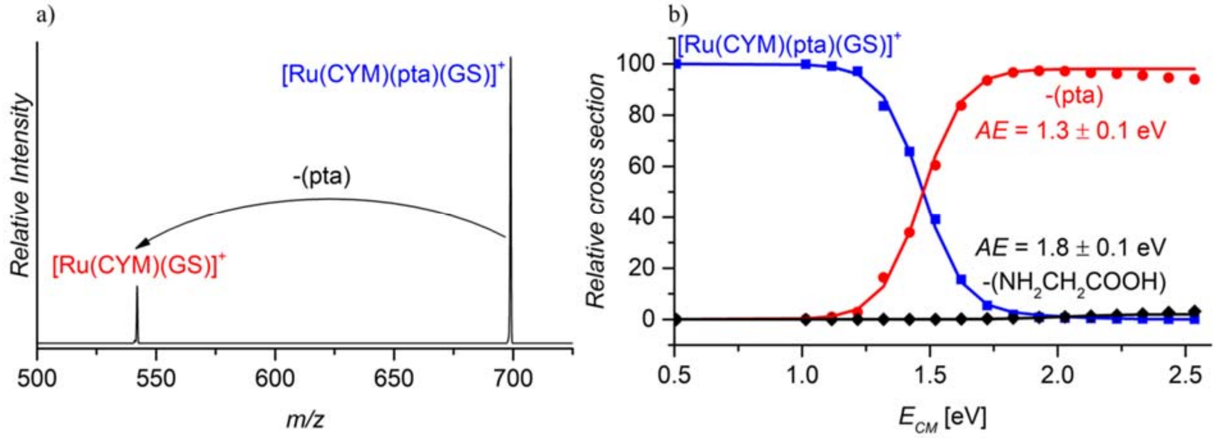

Figure S10. a) The CID spectrum of mass-selected ion $[Ru(CYM)(pta)(GS)]^+$ (E_{coll} 1.3 eV, c.m.) and b) the breakdown curve as function of collision energy.

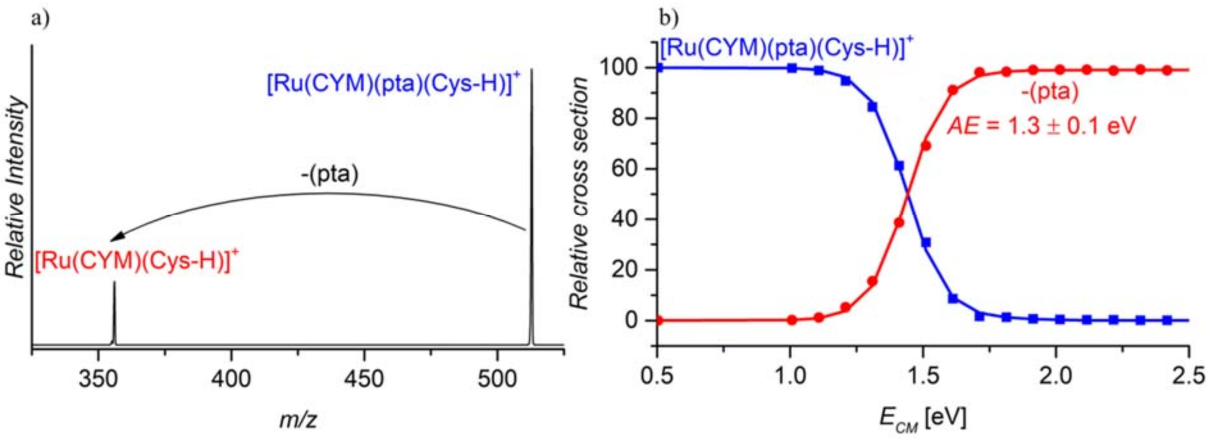

Figure S11. a) The CID spectrum of mass-selected ion [Ru(CYM)(pta)(Cys-H)]$^+$ (E_{coll} 1.3 eV, c.m.) and b) the breakdown curve as function of collision energy.

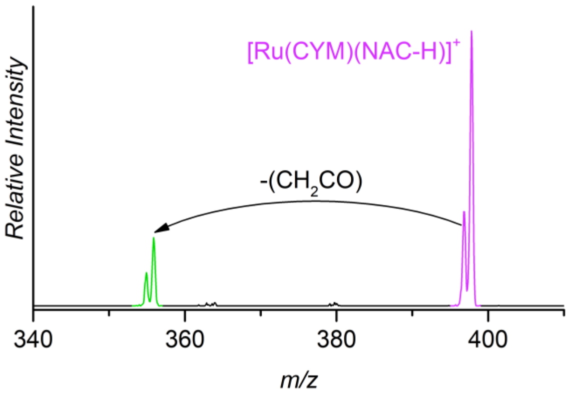

Figure S12. MS/MS/MS experiment of mass-selected ion [Ru(CYM)(NAC-H)]$^+$.

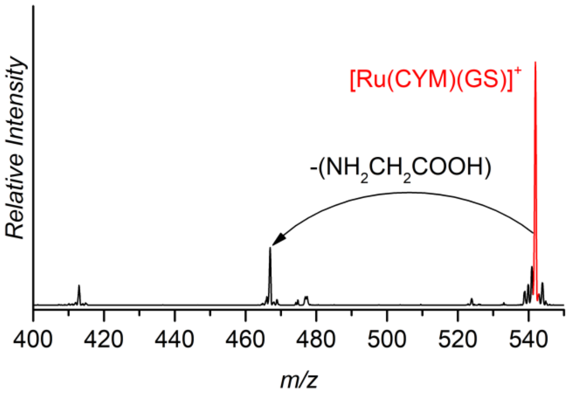

Figure S13. MS/MS/MS experiment of mass-selected ion [Ru(CYM)(GS)]$^+$.

IRPD and theoretical IR spectra

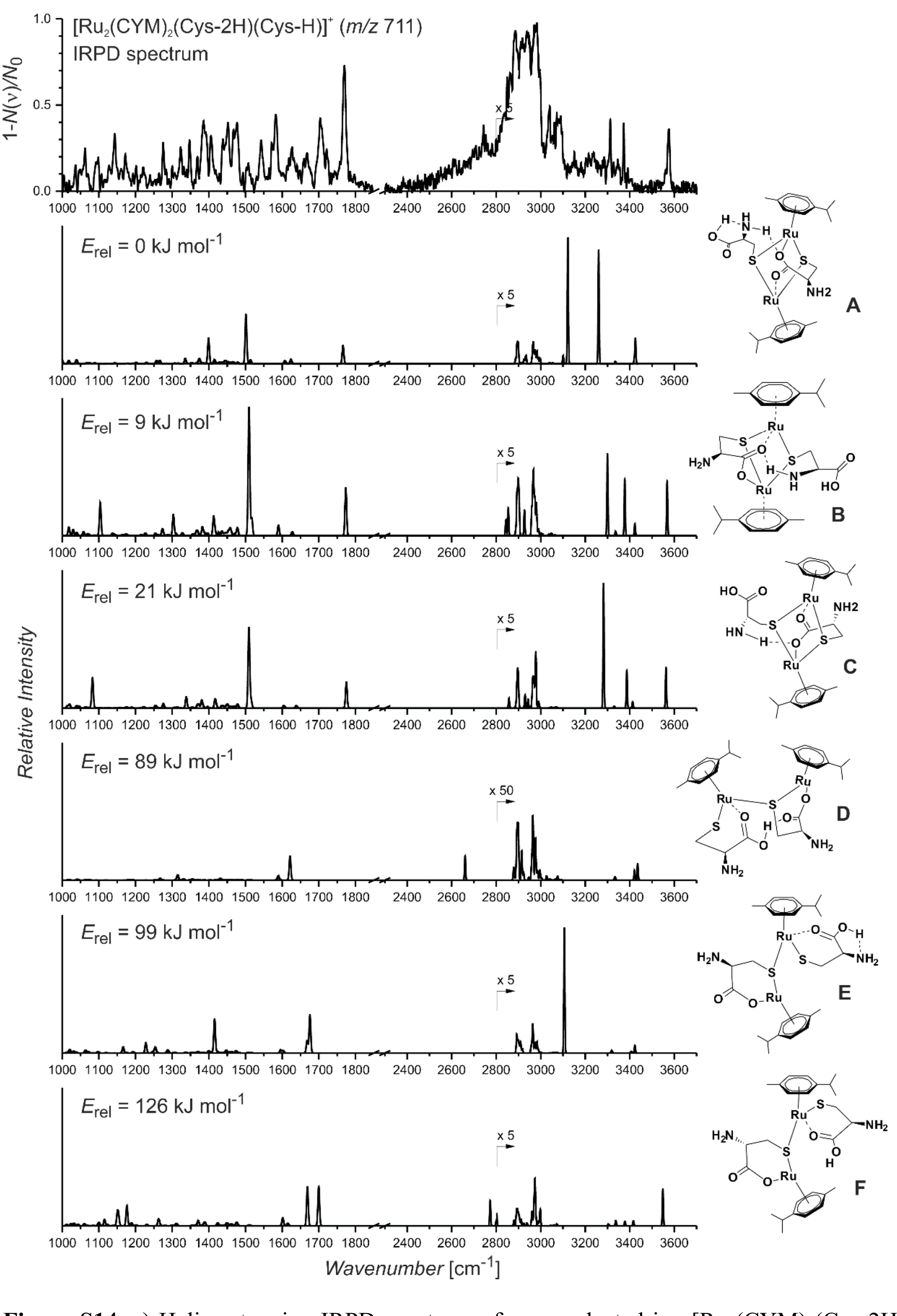

Figure S14. a) Helium tagging IRPD spectrum of mass-selected ion $[Ru_2(CYM)_2(Cys\text{-}2H)(Cys\text{-}H)]^+$ (*m/z* 711). b) Theoretical IR spectra (B3LYP-D3/6-31G**:SDD(Ru)) of possible isomers of $[Ru_2(CYM)_2(Cys\text{-}2H)(Cys\text{-}H)]^+$. The relative energies refer to energies at 0 K. The geometries and energetics of the optimised structures can be found in Table S3.

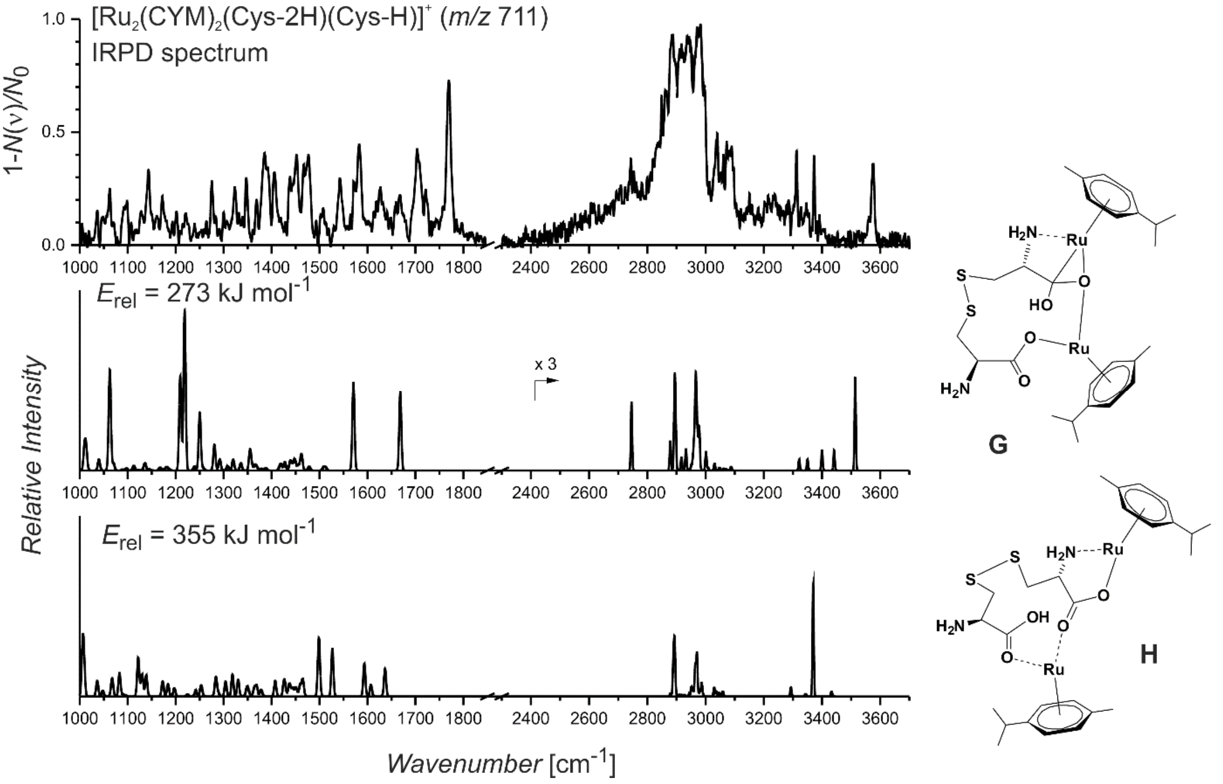

Figure S15. a) Helium tagging IRPD spectrum of mass-selected ion $[Ru_2(CYM)_2(Cys\text{-}2H)(Cys\text{-}H)]^+$ (*m/z* 711). b) Theoretical IR spectra (B3LYP-D3/6-31G**:SDD(Ru)) of possible isomers of $[Ru_2(CYM)_2(cystine)(Cys\text{-}H)]^+$. The relative energies refer to energies at 0 K and are given relative to the isomer **A** in Figure S12. The geometries and energetics of the optimised structures can be found in Table S3.

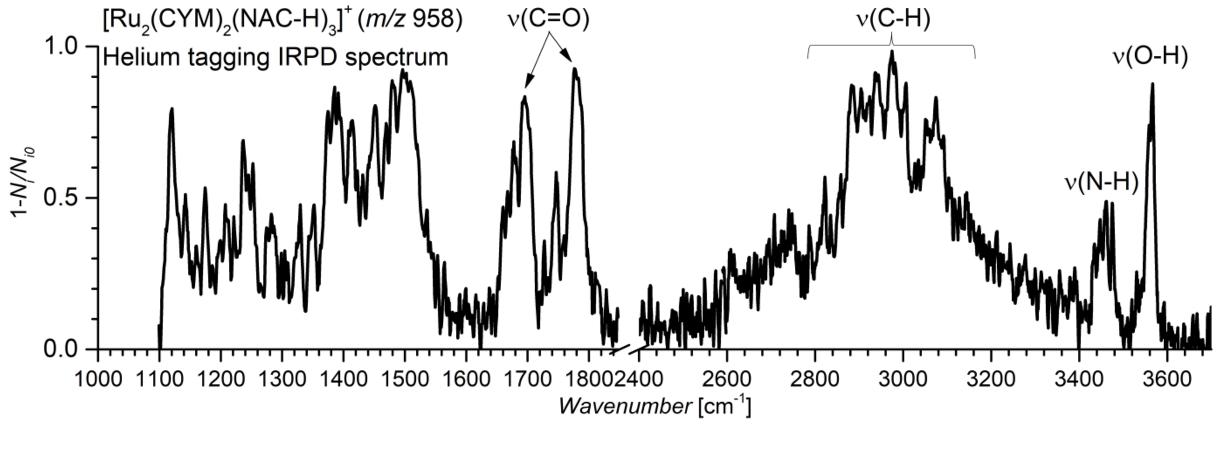

Figure S16. Helium tagging IRPD spectrum of mass-selected ions $[Ru_2(CYM)_2(NAC\text{-}H)_3]^+$ (*m/z* 958).

Computational results

Table S3. XYZ coordinates and energies of optimised structures of $[Ru_2(CYM)_2(Cys\text{-}2H)(Cys\text{-}H)]^{+}$. The calculations were perfomed at the B3LYP-D3/6-31G**:SDD(Ru) level of theory using Gaussian 16 workpackage.

A (Cys3)

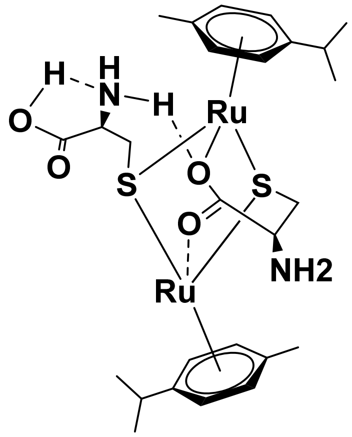

Charge = 1 Multiplicity = 1
Standard orientation:

Center Number	Atomic Number	Atomic Type	Coordinates (Angstroms) X	Y	Z
1	6	0	-3.687857	0.016618	-1.212205
2	6	0	-3.669362	-1.408458	-1.046909
3	6	0	-3.706092	-1.981558	0.241901
4	6	0	-3.836293	-1.100738	1.374990
5	6	0	-3.910564	0.293977	1.200234
6	6	0	-3.816440	0.895233	-0.103680
7	44	0	-2.060340	-0.426316	0.178255
8	16	0	-0.646508	1.327061	1.111453
9	6	0	-0.267855	0.830702	2.856176
10	6	0	-0.084614	-0.699497	3.050761
11	7	0	0.894423	-0.921267	4.100032
12	6	0	-3.542727	-3.459278	0.462538
13	6	0	-3.857123	2.406003	-0.239636
14	6	0	-3.069233	2.936570	-1.444117
15	44	0	1.426933	0.713902	-0.062729
16	8	0	2.643632	-1.305423	-3.207856
17	6	0	2.261909	-2.070796	-2.344359
18	6	0	0.825450	-2.629410	-2.301041
19	6	0	-0.176645	-1.522504	-2.648851
20	16	0	-0.383585	-0.055213	-1.523226
21	6	0	3.060640	1.861092	0.970438
22	6	0	3.706053	0.811743	0.255732
23	6	0	3.434655	0.674548	-1.133612
24	6	0	2.521944	1.540354	-1.788863
25	6	0	1.877414	2.611413	-1.099280
26	6	0	2.166322	2.739319	0.294280
27	6	0	4.663626	-0.171712	0.898539
28	6	0	4.414680	-0.419938	2.389592
29	6	0	0.910929	3.530789	-1.793947
30	8	0	1.394412	-1.127755	1.182176
31	6	0	0.241382	-1.300744	1.672784
32	8	0	-0.703864	-1.901358	1.049654
33	6	0	6.104210	0.323716	0.640819
34	8	0	3.036500	-2.516127	-1.357531
35	1	0	0.784023	-3.334805	-3.142319
36	6	0	-5.326205	2.873492	-0.272612
37	1	0	-3.540254	-2.046503	-1.914660
38	1	0	-3.549022	0.427094	-2.205272
39	1	0	-3.949259	0.938626	2.073314
40	1	0	-3.387966	2.808096	0.667421
41	1	0	-3.818947	-1.519669	2.375667
42	1	0	4.525931	-1.119913	0.366480
43	1	0	3.395730	-0.779068	2.545881
44	1	0	5.106866	-1.184052	2.753945
45	1	0	4.583521	0.479309	2.992568
46	1	0	6.820999	-0.403916	1.032148
47	1	0	6.284270	1.280960	1.142118
48	1	0	6.301340	0.453553	-0.427433
49	1	0	3.206691	1.971139	2.037790
50	1	0	1.629483	3.487850	0.869990
51	1	0	2.269428	1.332501	-2.823726
52	1	0	0.378356	3.000688	-2.587316
53	1	0	0.175124	3.930015	-1.091354

54	1	0	1.447485	4.373528	-2.244021
55	1	0	3.870751	-0.142218	-1.691960
56	1	0	-4.467056	-3.884630	0.867914
57	1	0	-3.304572	-3.977656	-0.468764
58	1	0	-2.736917	-3.642206	1.179222
59	1	0	-3.022886	4.028242	-1.400464
60	1	0	-2.048232	2.545565	-1.457714
61	1	0	-3.550755	2.674063	-2.392335
62	1	0	-5.879532	2.533194	0.608376
63	1	0	-5.373588	3.965960	-0.300978
64	1	0	-5.835680	2.489256	-1.162845
65	1	0	0.659921	1.346099	3.118084
66	1	0	-1.057770	1.212075	3.507354
67	1	0	-1.038305	-1.133394	3.364321
68	1	0	0.934010	-1.901688	4.364391
69	1	0	1.820990	-0.655114	3.777294
70	1	0	-1.179994	-1.943909	-2.745523
71	1	0	0.119671	-1.092892	-3.608003
72	1	0	2.421307	-3.081151	-0.812490
73	7	0	0.630876	-3.371749	-1.060289
74	1	0	0.148691	-2.823751	-0.344823
75	1	0	0.135757	-4.243813	-1.204828

Low frequencies --- -6.9918 -3.9681 -0.0016 -0.0015 -0.0014 5.1207

Low frequencies --- 14.9252 22.8554 33.2855

Zero-point correction= 0.625056 (Hartree/Particle)

Thermal correction to Energy= 0.665197

Thermal correction to Enthalpy= 0.666142

Thermal correction to Gibbs Free Energy= 0.554193

Sum of electronic and zero-point Energies= -2410.411735

Sum of electronic and thermal Energies= -2410.371594

Sum of electronic and thermal Enthalpies= -2410.370650

Sum of electronic and thermal Free Energies= -2410.482599

B (Cys1)

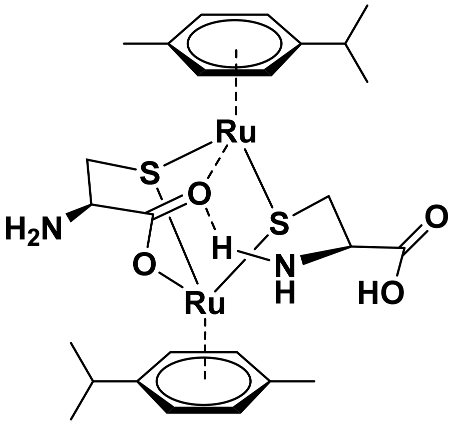

Charge = 1 Multiplicity = 1

Standard orientation:

Center Number	Atomic Number	Atomic Type	Coordinates (Angstroms) X	Y	Z
1	6	0	3.767140	-0.889043	-0.251657
2	6	0	3.915209	-0.407673	1.096750
3	6	0	3.842731	0.964196	1.397962
4	6	0	3.652826	1.942994	0.356990
5	6	0	3.559861	1.491502	-0.976936
6	6	0	3.591087	0.087984	-1.270219
7	44	0	2.022427	0.375022	0.216794
8	16	0	0.336879	0.056084	-1.488095
9	6	0	-0.090167	1.556235	-2.485169
10	6	0	-0.420814	2.916821	-1.853504
11	6	0	-1.799177	3.026778	-1.196006
12	8	0	-2.758120	2.330830	-1.864243
13	6	0	3.501256	3.394559	0.712121
14	6	0	3.816199	-2.381570	-0.522769
15	6	0	2.981846	-2.817839	-1.733743
16	8	0	0.752518	1.693682	1.456634
17	6	0	-0.379512	1.158374	1.688770
18	6	0	-0.511987	0.415780	3.016990
19	7	0	-1.894979	0.029242	3.283391
20	8	0	-1.331886	1.120202	0.857261
21	44	0	-1.414285	-0.844712	-0.092587
22	16	0	0.633879	-1.420234	1.130631
23	6	0	0.386109	-0.849318	2.887281
24	6	0	-1.815475	-2.954640	-0.630524
25	6	0	-2.438274	-2.676551	0.633780
26	6	0	-3.343224	-1.608493	0.784782
27	6	0	-3.723727	-0.796731	-0.339746
28	6	0	-3.109946	-1.050567	-1.583817
29	6	0	-2.142256	-2.099617	-1.716783
30	6	0	-0.831724	-4.082397	-0.781889

	31 6 0 -4.801673 0.262359 -0.205626<br>32 6 0 -4.589016 1.242691 0.958608<br>33 6 0 -6.166569 -0.450383 -0.091481<br>34 6 0 5.286463 -2.825090 -0.660868<br>35 7 0 0.653881 3.434016 -1.025232<br>36 8 0 -2.051237 3.742408 -0.255532<br>37 1 0 -0.515702 3.580518 -2.728863<br>38 1 0 3.343918 2.204891 -1.763742<br>39 1 0 3.409216 -0.233563 -2.288860<br>40 1 0 3.992663 -1.129122 1.905111<br>41 1 0 3.391651 -2.867783 0.365059<br>42 1 0 3.857789 1.291204 2.432328<br>43 1 0 -4.805997 0.828763 -1.143945<br>44 1 0 -3.626913 1.753633 0.890501<br>45 1 0 -5.387989 1.990436 0.957906<br>46 1 0 -4.639327 0.724247 1.921990<br>47 1 0 -6.972525 0.288911 -0.067118<br>48 1 0 -6.222438 -1.039071 0.830399<br>49 1 0 -6.343801 -1.122357 -0.936592<br>50 1 0 -3.710001 -1.370596 1.774293<br>51 1 0 -2.138358 -3.245999 1.507780<br>52 1 0 -1.623940 -2.227557 -2.661760<br>53 1 0 -0.156287 -3.905002 -1.622069<br>54 1 0 -0.230405 -4.200911 0.122731<br>55 1 0 -1.367019 -5.021029 -0.964679<br>56 1 0 -3.322590 -0.404187 -2.427856<br>57 1 0 4.482813 3.821185 0.948140<br>58 1 0 3.057569 3.950785 -0.114351<br>59 1 0 2.860670 3.504270 1.591572<br>60 1 0 2.954622 -3.909924 -1.786671<br>61 1 0 1.956142 -2.445035 -1.666264<br>62 1 0 3.414034 -2.461128 -2.674862<br>63 1 0 5.874978 -2.555669 0.221829<br>64 1 0 5.344146 -3.910128 -0.788167<br>65 1 0 5.751958 -2.356333 -1.534573<br>66 1 0 -0.069799 -1.673098 3.441969<br>67 1 0 1.388392 -0.666016 3.280108<br>68 1 0 -0.072297 1.062537 3.791224<br>69 1 0 -2.028048 -0.115757 4.280444<br>70 1 0 -2.521696 0.774163 2.992980<br>71 1 0 0.791515 1.697047 -3.115897<br>72 1 0 -0.917463 1.236439 -3.122195<br>73 1 0 0.654502 2.924836 -0.141268<br>74 1 0 0.428449 4.395168 -0.777822<br>75 1 0 -3.598394 2.562050 -1.434380<br>---<br>Low frequencies --- -5.7868 -0.0008 -0.0006 0.0005 4.5637 6.7313<br>Low frequencies --- 23.7977 27.3928 38.5098<br><br>Zero-point correction= 0.624823 (Hartree/Particle)<br>Thermal correction to Energy= 0.664954<br>Thermal correction to Enthalpy= 0.665898<br>Thermal correction to Gibbs Free Energy= 0.555365<br>Sum of electronic and zero-point Energies= -2410.408407<br>Sum of electronic and thermal Energies= -2410.368276<br>Sum of electronic and thermal Enthalpies= -2410.367332<br>Sum of electronic and thermal Free Energies= -2410.477865
**C (Cys6)**	Charge = 1 Multiplicity = 1<br>Standard orientation:<br>---<br>Center Atomic Atomic Coordinates (Angstroms)<br>Number Number Type X Y Z<br>---<br>1 6 0 -2.236446 -2.221052 -1.586280<br>2 6 0 -3.028523 -2.151650 -0.391307<br>3 6 0 -3.673938 -0.953677 -0.028560<br>4 6 0 -3.531172 0.180746 -0.911083<br>5 6 0 -2.828473 0.082032 -2.128608<br>6 6 0 -2.152081 -1.131835 -2.493990<br>7 44 0 -1.470128 -0.509347 -0.476018

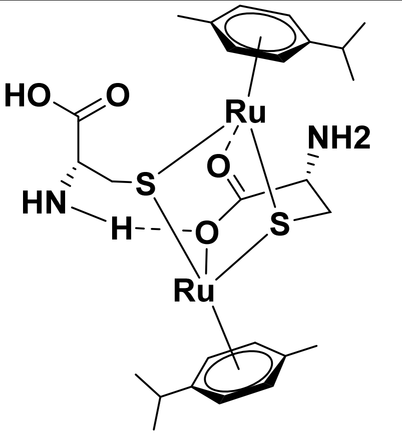

8	8	0	-1.454831	0.488166	1.481763
9	6	0	-0.323609	0.392372	2.041976
10	8	0	0.671840	1.141354	1.768375
11	44	0	2.026049	0.166282	0.375524
12	16	0	0.441853	0.648771	-1.374297
13	6	0	0.288341	2.428046	-1.867045
14	6	0	-0.325578	3.476403	-0.928544
15	7	0	0.421041	3.618430	0.299826
16	6	0	-4.437817	-0.766340	1.268664
17	6	0	-5.921331	-0.473405	0.956677
18	6	0	-1.359465	-1.225775	-3.767632
19	6	0	3.706516	0.295616	-1.015785
20	6	0	3.676391	1.521684	-0.270947
21	6	0	3.663268	1.506282	1.141173
22	6	0	3.751226	0.228509	1.803736
23	6	0	3.818629	-0.969522	1.067004
24	6	0	3.772274	-0.968705	-0.371937
25	6	0	3.485600	2.772661	1.930088
26	6	0	3.793832	-2.285244	-1.126392
27	6	0	5.254513	-2.744740	-1.307344
28	16	0	0.485860	-1.760042	0.386779
29	6	0	0.018325	-2.055113	2.159769
30	6	0	-0.110110	-0.764490	3.023746
31	7	0	-1.166289	-0.954039	4.008215
32	6	0	3.047982	-2.238663	-2.466451
33	6	0	-1.838404	3.288518	-0.747674
34	8	0	-2.430992	3.243068	0.303480
35	8	0	-2.481260	3.261612	-1.953000
36	6	0	-4.317838	-1.943445	2.243016
37	1	0	-0.247674	4.416511	-1.497037
38	1	0	3.556324	2.465323	-0.791838
39	1	0	3.605506	0.339532	-2.093670
40	1	0	3.814085	-1.919792	1.592543
41	1	0	3.279755	-3.013650	-0.486353
42	1	0	3.688827	0.190807	2.886173
43	1	0	-4.000619	0.123139	1.741148
44	1	0	-3.278485	-2.188436	2.479252
45	1	0	-4.820181	-1.697278	3.182440
46	1	0	-4.798667	-2.844203	1.845562
47	1	0	-6.463645	-0.272817	1.884875
48	1	0	-6.392913	-1.332219	0.467363
49	1	0	-6.042248	0.397254	0.305739
50	1	0	-3.049324	-3.007400	0.271884
51	1	0	-1.670947	-3.124291	-1.792300
52	1	0	-2.703443	0.967050	-2.742415
53	1	0	-0.878427	-0.273080	-4.001483
54	1	0	-0.581426	-1.988979	-3.690967
55	1	0	-2.021724	-1.491169	-4.599413
56	1	0	-3.921375	1.140028	-0.590496
57	1	0	4.464113	3.208354	2.162390
58	1	0	2.895000	3.494997	1.361109
59	1	0	2.966218	2.570415	2.869494
60	1	0	2.983278	-3.246620	-2.885444
61	1	0	2.034170	-1.846115	-2.345180
62	1	0	3.572059	-1.618181	-3.201534
63	1	0	5.776363	-2.821839	-0.348278
64	1	0	5.284641	-3.726397	-1.789117
65	1	0	5.807155	-2.040468	-1.938516
66	1	0	-0.945928	-2.567261	2.134401
67	1	0	0.749336	-2.740012	2.595883
68	1	0	0.835606	-0.588746	3.542606
69	1	0	-1.083409	-0.269600	4.755143
70	1	0	-2.066443	-0.795992	3.561083
71	1	0	1.316490	2.728712	-2.083503
72	1	0	-0.267037	2.409011	-2.807452
73	1	0	0.343703	2.765690	0.856255
74	1	0	0.017281	4.366318	0.857824
75	1	0	-3.433883	3.306990	-1.764990

Low frequencies --- -4.8667 -3.3099 -0.0015 -0.0007 0.0000 2.6795

Low frequencies --- 24.0300 27.1582 34.7419
Zero-point correction= 0.624753 (Hartree/Particle)
Thermal correction to Energy= 0.665102
Thermal correction to Enthalpy= 0.666046
Thermal correction to Gibbs Free Energy= 0.553963
Sum of electronic and zero-point Energies= -2410.403688
Sum of electronic and thermal Energies= -2410.363339
Sum of electronic and thermal Enthalpies= -2410.362395
Sum of electronic and thermal Free Energies= -2410.474478

D (Cys5)

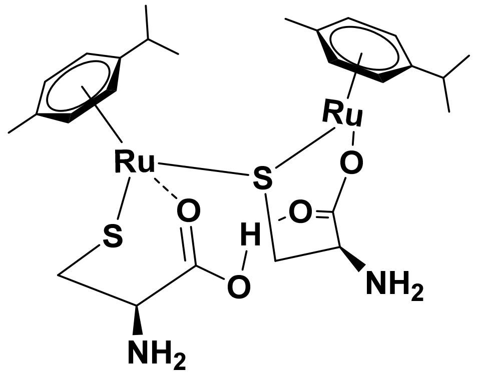

Charge = 1 Multiplicity = 1
Standard orientation:

Center Number	Atomic Number	Atomic Type	X	Y	Z
1	6	0	2.800042	-2.080374	-0.618636
2	6	0	2.685127	-1.637420	-1.957738
3	6	0	3.169319	-0.322487	-2.233930
4	6	0	3.809376	0.450151	-1.218752
5	6	0	4.022529	-0.054962	0.109954
6	6	0	3.480342	-1.320127	0.394289
7	44	0	1.824690	-0.070991	-0.550723
8	8	0	1.413434	1.508827	0.641116
9	6	0	0.607107	2.515481	0.491160
10	8	0	0.164141	3.124897	1.476244
11	6	0	2.005933	-2.445501	-3.029743
12	6	0	4.699540	0.819887	1.146222
13	6	0	6.220829	0.566125	1.101037
14	16	0	-0.162117	0.248865	-1.758657
15	6	0	-0.536621	2.069155	-1.799648
16	6	0	0.321479	3.012609	-0.943680
17	7	0	1.549329	3.351662	-1.666897
18	44	0	-1.647839	-0.736749	-0.085128
19	16	0	-3.416596	-0.036841	-1.560179
20	6	0	-4.543132	0.698719	-0.300922
21	6	0	-1.252447	-1.662696	1.991550
22	6	0	-0.263246	-2.164076	1.121171
23	6	0	-0.642223	-2.702405	-0.155191
24	6	0	-1.994364	-2.893148	-0.536535
25	6	0	-2.990359	-2.449961	0.395915
26	6	0	-2.629846	-1.830709	1.608443
27	6	0	-0.942643	-0.982994	3.311850
28	6	0	-1.138546	-2.001419	4.455334
29	6	0	-2.380122	-3.508554	-1.852154
30	8	0	-1.863554	1.184236	0.799792
31	6	0	-2.699151	2.112885	0.782905
32	6	0	-4.108680	2.083930	0.181604
33	1	0	-4.777760	2.401233	0.993927
34	6	0	0.439947	-0.325627	3.369682
35	6	0	4.130525	0.647322	2.560677
36	1	0	-4.036229	-2.504689	0.112950
37	1	0	-3.405347	-1.435328	2.256529
38	1	0	0.781656	-2.078653	1.385850
39	1	0	-1.692359	-0.189650	3.428282
40	1	0	0.129848	-2.977545	-0.862553
41	1	0	4.515120	1.857524	0.840997
42	1	0	4.344891	-0.347084	2.968016
43	1	0	4.597774	1.373218	3.231495
44	1	0	3.051489	0.817195	2.570565
45	1	0	6.732283	1.245262	1.788873
46	1	0	6.631371	0.725677	0.098835
47	1	0	6.451604	-0.460697	1.404112
48	1	0	4.137990	1.457875	-1.453349
49	1	0	3.008767	0.113556	-3.215203
50	1	0	2.361574	-3.034207	-0.341657
51	1	0	1.536942	-3.344072	-2.622030
52	1	0	1.242036	-1.850220	-3.538779
53	1	0	2.740819	-2.764975	-3.776047
54	1	0	3.512446	-1.708917	1.405492
55	1	0	-2.657479	-4.559149	-1.710974

56	1	0	-3.233132	-2.978108	-2.282934
57	1	0	-1.551771	-3.464226	-2.563648
58	1	0	0.554882	0.198503	4.322479
59	1	0	1.243375	-1.070343	3.318336
60	1	0	0.575524	0.400931	2.566325
61	1	0	-0.407674	-2.813765	4.378903
62	1	0	-0.998210	-1.509015	5.421657
63	1	0	-2.139537	-2.443996	4.442517
64	1	0	-0.401996	2.366899	-2.842152
65	1	0	-1.594545	2.148227	-1.562106
66	1	0	-0.253264	3.937514	-0.828187
67	1	0	2.088784	4.043158	-1.151722
68	1	0	2.129401	2.523422	-1.761838
69	7	0	-4.242482	3.054912	-0.900688
70	1	0	-3.908562	3.972632	-0.624576
71	1	0	-3.736878	2.742062	-1.725493
72	8	0	-2.378416	3.282545	1.270092
73	1	0	-5.522057	0.811508	-0.775008
74	1	0	-4.655736	0.015217	0.546022
75	1	0	-1.375131	3.318334	1.460853

Low frequencies --- -3.6722 -1.3118 0.0011 0.0012 0.0014 5.9417

Low frequencies --- 19.3365 28.8707 34.9809

Zero-point correction= 0.624392 (Hartree/Particle)

Thermal correction to Energy= 0.664399

Thermal correction to Enthalpy= 0.665343

Thermal correction to Gibbs Free Energy= 0.553988

Sum of electronic and zero-point Energies= -2410.377872

Sum of electronic and thermal Energies= -2410.337865

Sum of electronic and thermal Enthalpies= -2410.336921

Sum of electronic and thermal Free Energies= -2410.448276

E (Cys4)

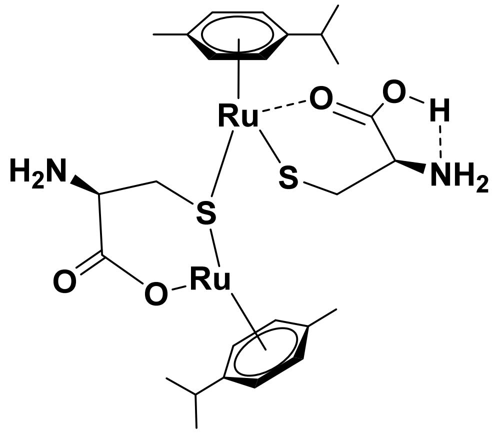

Charge = 1 Multiplicity = 1

Standard orientation:

Center Number	Atomic Number	Atomic Type	Coordinates (Angstroms) X	Y	Z
1	6	0	3.363774	1.719151	0.425304
2	6	0	3.080473	1.679317	-0.966373
3	6	0	3.380747	0.528350	-1.767719
4	6	0	3.891278	-0.644342	-1.178471
5	6	0	4.161387	-0.616242	0.238196
6	6	0	3.947642	0.540836	1.009673
7	44	0	2.039576	0.004021	-0.013067
8	16	0	0.816442	0.325957	2.027637
9	6	0	4.078994	-1.919774	-1.950378
10	6	0	3.101486	2.938032	1.289716
11	6	0	1.890627	3.760240	0.832660
12	16	0	0.083159	0.495145	-1.425919
13	6	0	0.007105	-0.783934	-2.764027
14	6	0	-0.346977	-2.209675	-2.327241
15	7	0	-0.745219	-2.960349	-3.521744
16	44	0	-2.168314	0.511880	-0.680599
17	8	0	-2.409332	-1.378645	-1.279081
18	6	0	-1.415730	-2.270088	-1.226887
19	8	0	-1.401152	-3.156930	-0.384059
20	6	0	-2.365839	2.741660	-0.702361
21	6	0	-3.452146	2.097375	-1.380736
22	6	0	-4.315939	1.167905	-0.716529
23	6	0	-4.036627	0.732095	0.594333
24	6	0	-2.860675	1.279594	1.219680
25	6	0	-2.088174	2.313451	0.618532
26	6	0	-1.502147	3.748987	-1.407691
27	6	0	-4.884134	-0.325388	1.275630
28	6	0	-4.062689	-1.506944	1.820263
29	6	0	-5.724079	0.339525	2.384707
30	8	0	1.525618	-2.147374	0.070817
31	6	0	1.349518	-2.738635	1.141972
32	6	0	0.225412	-2.459738	2.136453
33	6	0	4.380427	3.796694	1.354296

34	1	0	4.480489	-1.532029	0.726795
35	1	0	4.111128	0.507227	2.081643
36	1	0	2.581688	2.517985	-1.436596
37	1	0	2.886457	2.563734	2.298399
38	1	0	3.112990	0.531061	-2.818977
39	1	0	-5.567981	-0.719885	0.514916
40	1	0	-3.403601	-1.190487	2.636165
41	1	0	-4.742235	-2.259481	2.230738
42	1	0	-3.458190	-1.973298	1.037878
43	1	0	-6.386673	-0.398692	2.845449
44	1	0	-6.340932	1.154182	1.993239
45	1	0	-5.079370	0.748422	3.171046
46	1	0	-5.113297	0.693425	-1.279749
47	1	0	-3.627089	2.325923	-2.427808
48	1	0	-1.196520	2.656569	1.130826
49	1	0	-0.488148	3.748698	-1.003704
50	1	0	-1.444583	3.535378	-2.477904
51	1	0	-1.924238	4.752192	-1.280553
52	1	0	-2.538583	0.895643	2.180443
53	1	0	5.145406	-2.150973	-2.046700
54	1	0	3.595341	-2.748062	-1.424657
55	1	0	3.650356	-1.846327	-2.952475
56	1	0	1.670039	4.537822	1.568975
57	1	0	2.075240	4.262537	-0.123345
58	1	0	1.012567	3.117206	0.731252
59	1	0	4.634589	4.189456	0.363788
60	1	0	4.231474	4.645610	2.027906
61	1	0	5.235542	3.219929	1.720103
62	1	0	-0.742372	-0.413398	-3.469861
63	1	0	0.971572	-0.785369	-3.277250
64	1	0	0.535506	-2.692169	-1.907526
65	1	0	-0.828968	-3.949583	-3.301981
66	1	0	-1.656633	-2.646781	-3.850309
67	8	0	2.145329	-3.720955	1.495889
68	1	0	1.870508	-3.919736	2.436148
69	1	0	-0.555563	-3.173365	1.838465
70	7	0	0.737994	-2.860443	3.452589
71	6	0	-0.377878	-1.057980	2.010643
72	1	0	-0.977675	-1.021370	1.098813
73	1	0	-1.061023	-0.915425	2.853747
74	1	0	1.225804	-2.077764	3.887305
75	1	0	-0.010305	-3.152117	4.072863

Low frequencies --- -2.5970 -0.0010 0.0002 0.0009 3.7594 7.7714

Low frequencies --- 21.5046 29.8071 34.6574

Zero-point correction= 0.624567 (Hartree/Particle)
Thermal correction to Energy= 0.665150
Thermal correction to Enthalpy= 0.666094
Thermal correction to Gibbs Free Energy= 0.552370
Sum of electronic and zero-point Energies= -2410.373850
Sum of electronic and thermal Energies= -2410.333267
Sum of electronic and thermal Enthalpies= -2410.332323
Sum of electronic and thermal Free Energies= -2410.446047

F (Cys2)

Charge = 1 Multiplicity = 1
Standard orientation:

Center Number	Atomic Number	Atomic Type	Coordinates (Angstroms) X	Y	Z
1	6	0	2.066277	-1.555137	-2.315782
2	6	0	3.040718	-0.527930	-2.551566
3	6	0	4.042666	-0.216382	-1.576037
4	6	0	4.024779	-0.801886	-0.294579
5	6	0	2.951365	-1.718178	-0.017845
6	6	0	2.048643	-2.149260	-1.031126
7	44	0	1.966808	0.083399	-0.795864
8	8	0	2.359057	1.965527	-0.274706
9	6	0	1.685333	3.083444	-0.001899
10	8	0	2.224374	3.997961	0.593793

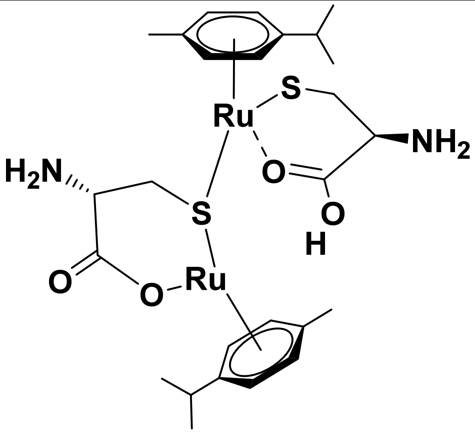

11	6	0	5.025011	-0.406593	0.776923
12	6	0	5.848545	-1.637085	1.201959
13	6	0	1.057455	-1.918769	-3.368079
14	16	0	-0.285363	0.574105	-1.328749
15	44	0	-2.093317	0.204063	0.338210
16	6	0	-3.670599	0.018915	-1.313782
17	6	0	-3.436670	1.383016	-0.967799
18	6	0	-3.552020	1.869289	0.364817
19	6	0	-3.929385	0.910780	1.368105
20	6	0	-4.103887	-0.449485	1.047860
21	6	0	-3.948476	-0.927079	-0.304124
22	6	0	-3.310043	3.312676	0.712711
23	6	0	-4.091410	-2.411319	-0.583051
24	6	0	-5.580944	-2.748621	-0.800144
25	6	0	-0.186451	2.391724	-1.631369
26	6	0	0.201086	3.210464	-0.404367
27	7	0	-0.236934	4.596298	-0.582603
28	16	0	-0.889664	0.995391	2.221719
29	6	0	0.603952	-0.023601	2.326967
30	6	0	0.352353	-1.510172	2.631685
31	7	0	1.611758	-2.179667	2.974584
32	6	0	-0.323463	-2.229003	1.478228
33	8	0	-1.116995	-1.754935	0.665665
34	8	0	-0.035061	-3.531405	1.424915
35	6	0	-3.226490	-2.903952	-1.751691
36	6	0	4.351677	0.269876	1.984723
37	1	0	-0.404583	-1.552828	3.437509
38	1	0	-3.973002	1.223652	2.406422
39	1	0	-4.292727	-1.163299	1.843503
40	1	0	-3.511295	-0.297058	-2.336771
41	1	0	-3.755225	-2.928428	0.325451
42	1	0	-3.124123	2.071118	-1.745121
43	1	0	5.705598	0.322766	0.322479
44	1	0	3.655239	-0.417991	2.474051
45	1	0	5.112153	0.556422	2.717067
46	1	0	3.806936	1.165955	1.676425
47	1	0	6.619934	-1.340785	1.918474
48	1	0	6.342095	-2.110347	0.347407
49	1	0	5.212579	-2.386160	1.686969
50	1	0	4.741437	0.588365	-1.782695
51	1	0	3.029413	0.008437	-3.495131
52	1	0	1.268514	-2.858112	-0.781124
53	1	0	0.136467	-2.297264	-2.918091
54	1	0	0.800946	-1.051847	-3.981133
55	1	0	1.470027	-2.694631	-4.022785
56	1	0	2.808190	-2.084490	0.997431
57	1	0	-4.257582	3.863209	0.685416
58	1	0	-2.897378	3.400892	1.721402
59	1	0	-2.611027	3.791226	0.020010
60	1	0	-3.277578	-3.994958	-1.815940
61	1	0	-3.579792	-2.512244	-2.711007
62	1	0	-2.181835	-2.605854	-1.621504
63	1	0	-5.962925	-2.248618	-1.696249
64	1	0	-5.709736	-3.826981	-0.931976
65	1	0	-6.193481	-2.434678	0.050512
66	1	0	0.524733	2.527089	-2.452697
67	1	0	-1.164692	2.722065	-1.983952
68	1	0	-0.348722	2.809517	0.453158
69	1	0	0.087268	5.124522	0.226095
70	1	0	0.257304	5.009800	-1.373064
71	1	0	1.210371	0.391245	3.138159
72	1	0	1.214711	0.060857	1.411458
73	1	0	2.010210	-1.728375	3.793655
74	1	0	1.453572	-3.153453	3.220092
75	1	0	-0.570059	-3.936989	0.719737

Low frequencies --- 0.0004 0.0009 0.0019 1.4989 4.6953 7.8766

Low frequencies --- 18.0633 19.1549 25.7330

Zero-point correction= 0.624051 (Hartree/Particle)

Thermal correction to Energy= 0.664609

Thermal correction to Enthalpy= 0.665553
Thermal correction to Gibbs Free Energy= 0.551501
Sum of electronic and zero-point Energies= -2410.363853
Sum of electronic and thermal Energies= -2410.323296
Sum of electronic and thermal Enthalpies= -2410.322352
Sum of electronic and thermal Free Energies= -2410.436404

G (oxCys6)

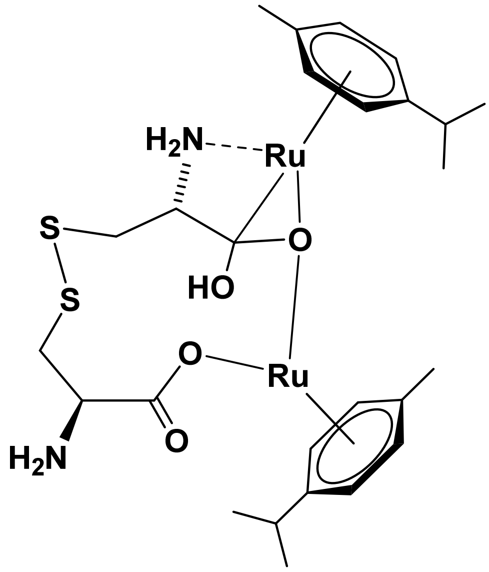

Charge = 1 Multiplicity = 1
Standard orientation:

Center Number	Atomic Number	Atomic Type	Coordinates (Angstroms) X	Y	Z
1	44	0	1.838865	-1.357150	-0.270396
2	6	0	3.016674	-3.034018	0.584603
3	6	0	3.509642	-2.725696	-0.716891
4	6	0	4.042231	-1.441751	-1.066070
5	6	0	3.951470	-0.379070	-0.151089
6	6	0	3.385282	-0.653216	1.144527
7	6	0	2.956278	-1.951286	1.517570
8	6	0	2.575095	-4.423289	0.961776
9	6	0	4.420376	1.026886	-0.484298
10	6	0	3.933807	1.512222	-1.858269
11	6	0	5.954731	1.111807	-0.366985
12	6	0	-0.088722	-1.285812	0.360831
13	8	0	0.235113	0.001358	-0.083578
14	8	0	-0.521194	-1.376707	1.653559
15	44	0	-0.850943	1.676241	-0.103003
16	6	0	-1.199661	3.112364	-1.727708
17	6	0	-1.989795	3.528633	-0.615011
18	6	0	-1.409087	3.670880	0.664077
19	6	0	-0.008530	3.439122	0.878077
20	6	0	0.805870	3.143805	-0.257995
21	6	0	0.214698	2.951740	-1.526361
22	6	0	-1.832589	2.846857	-3.063793
23	6	0	0.567425	3.523519	2.279496
24	6	0	1.144840	4.936980	2.499649
25	6	0	1.608404	2.433825	2.578274
26	8	0	-2.401121	0.418871	0.366999
27	6	0	-3.614802	0.574311	-0.128392
28	8	0	-3.890851	1.297874	-1.078819
29	6	0	-4.730455	-0.166245	0.626133
30	1	0	-4.946318	0.503457	1.485482
31	7	0	-5.860182	-0.366807	-0.267975
32	1	0	-6.720444	-0.513219	0.249883
33	1	0	-5.969885	0.434685	-0.880963
34	7	0	0.368147	-1.991645	-1.765540
35	1	0	0.200561	-1.275501	-2.470166
36	1	0	0.510046	-2.874262	-2.250860
37	6	0	-0.769026	-2.058039	-0.765383
38	1	0	-1.664611	-1.556362	-1.148935
39	6	0	-1.083072	-3.520844	-0.472965
40	1	0	-3.065303	3.578537	-0.727967
41	1	0	0.825994	2.597200	-2.350263
42	1	0	1.855898	2.912996	-0.125960
43	1	0	-2.047825	3.857231	1.521575
44	1	0	-0.271063	3.383707	2.972905
45	1	0	3.989817	1.690135	0.277765
46	1	0	2.848980	1.396997	-1.949751
47	1	0	4.194351	2.565334	-2.003543
48	1	0	4.398876	0.949024	-2.673579
49	1	0	6.295761	2.137441	-0.537967
50	1	0	6.434243	0.466554	-1.110480
51	1	0	6.297486	0.798391	0.623584
52	1	0	4.427553	-1.280953	-2.065762
53	1	0	3.494674	-3.505448	-1.473359
54	1	0	2.514133	-2.108781	2.496298
55	1	0	1.755064	-4.392289	1.684149
56	1	0	2.245939	-4.987494	0.084762
57	1	0	3.406099	-4.973626	1.418021
58	1	0	3.263624	0.162171	1.848782

59	1	0	-1.966050	3.795190	-3.598011
60	1	0	-1.202271	2.199087	-3.678669
61	1	0	-2.807535	2.375893	-2.928490
62	1	0	1.885940	2.469932	3.635377
63	1	0	1.218037	1.436526	2.354023
64	1	0	2.525580	2.587300	1.998898
65	1	0	0.392305	5.712217	2.327366
66	1	0	1.507896	5.037150	3.526492
67	1	0	1.986213	5.122352	1.823069
68	1	0	-1.434723	-1.041070	1.674124
69	16	0	-2.482292	-3.881920	0.670723
70	1	0	-1.284877	-4.034741	-1.419236
71	1	0	-0.223365	-4.005076	0.004440
72	6	0	-4.315974	-1.510293	1.220046
73	1	0	-3.402960	-1.434418	1.809788
74	1	0	-5.108267	-1.877259	1.878207
75	16	0	-4.082093	-2.777542	-0.119802

Low frequencies --- -6.1012 -4.2446 0.0007 0.0008 0.0012 1.8745

Low frequencies --- 13.4987 18.8828 28.0495

Zero-point correction= 0.623386 (Hartree/Particle)

Thermal correction to Energy= 0.663472

Thermal correction to Enthalpy= 0.664416

Thermal correction to Gibbs Free Energy= 0.550645

Sum of electronic and zero-point Energies= -2410.307906

Sum of electronic and thermal Energies= -2410.267821

Sum of electronic and thermal Enthalpies= -2410.266876

Sum of electronic and thermal Free Energies= -2410.380648

H (oxCys2)

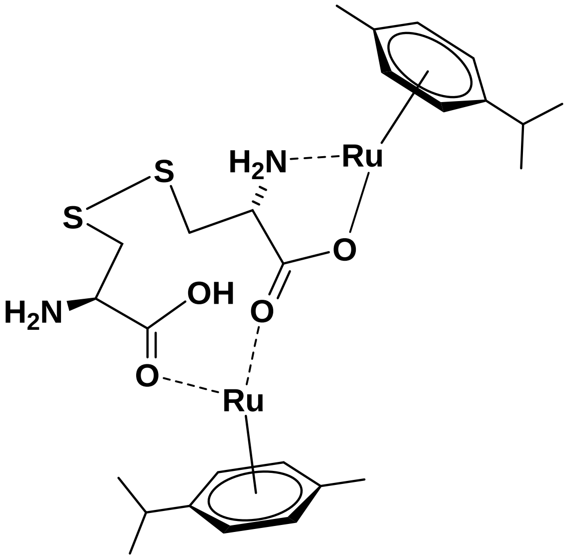

Charge = 1 Multiplicity = 1

Standard orientation:

Center Number	Atomic Number	Atomic Type	Coordinates (Angstroms) X	Y	Z
1	44	0	2.370714	0.663782	0.667699
2	6	0	3.744162	2.403852	1.241515
3	6	0	4.487975	1.202324	1.045359
4	6	0	4.430599	0.455435	-0.170191
5	6	0	3.581065	0.856314	-1.237246
6	6	0	2.782293	2.022664	-1.017138
7	6	0	2.861301	2.778434	0.187984
8	6	0	3.882321	3.227242	2.492866
9	6	0	3.503191	0.111153	-2.557381
10	6	0	3.567049	-1.415135	-2.404833
11	6	0	4.604490	0.625293	-3.506918
12	8	0	0.303828	0.697331	0.827313
13	6	0	-0.265074	-0.272658	1.467181
14	8	0	-1.502615	-0.333789	1.693988
15	6	0	0.639071	-1.461061	1.757843
16	7	0	2.006273	-0.974217	2.072452
17	44	0	-2.618747	0.389865	0.047700
18	6	0	-3.017652	2.576953	-0.141179
19	6	0	-3.626296	2.051578	1.043043
20	6	0	-4.553542	0.968391	1.010131
21	6	0	-4.858071	0.318808	-0.216561
22	6	0	-4.217440	0.819073	-1.398061
23	6	0	-3.347139	1.943625	-1.375571
24	6	0	-5.794555	-0.856383	-0.284358
25	6	0	-2.072143	3.763032	-0.063316
26	6	0	-0.786392	3.574247	-0.883988
27	6	0	-2.824589	5.043318	-0.478091
28	8	0	-1.914602	-1.356113	-1.010328
29	6	0	-0.827763	-1.715203	-1.473486
30	8	0	0.251762	-0.937527	-1.519087
31	6	0	-0.589189	-3.107351	-2.051033
32	7	0	0.118471	-3.107939	-3.321705
33	1	0	0.997623	-2.603141	-3.253427
34	1	0	-0.435660	-2.685491	-4.060430
35	1	0	-1.579376	-3.548393	-2.199859
36	1	0	0.116980	-0.101068	-1.020990

37	1	0	2.664034	-1.748769	1.999142
38	1	0	2.052054	-0.641933	3.037029
39	6	0	0.076149	-2.537919	2.681492
40	16	0	0.500045	-4.238915	2.091089
41	1	0	-4.962741	0.585439	1.939832
42	1	0	-4.371073	0.293721	-2.336016
43	1	0	-2.880734	2.270041	-2.298288
44	1	0	-3.336098	2.463458	2.004697
45	1	0	-1.778655	3.863867	0.989068
46	1	0	2.529087	0.358382	-2.999821
47	1	0	2.808642	-1.764067	-1.697867
48	1	0	3.400793	-1.896835	-3.374161
49	1	0	4.547948	-1.749781	-2.051547
50	1	0	4.520794	0.145318	-4.487081
51	1	0	5.597574	0.400386	-3.103437
52	1	0	4.535446	1.707824	-3.649844
53	1	0	5.016151	-0.453037	-0.252939
54	1	0	5.112847	0.833905	1.854202
55	1	0	2.205953	3.634210	0.317217
56	1	0	2.952089	3.751713	2.727019
57	1	0	4.154937	2.605792	3.350265
58	1	0	4.669589	3.980640	2.366148
59	1	0	2.066115	2.322596	-1.776652
60	1	0	-6.804055	-0.519319	-0.548729
61	1	0	-5.471352	-1.572360	-1.044777
62	1	0	-5.850384	-1.374389	0.676123
63	1	0	-0.129839	4.439811	-0.746054
64	1	0	-0.254924	2.674939	-0.563187
65	1	0	-0.996269	3.499703	-1.956775
66	1	0	-3.722183	5.195086	0.128911
67	1	0	-2.180418	5.919997	-0.359085
68	1	0	-3.131560	4.989739	-1.528363
69	1	0	0.739274	-1.888475	0.752594
70	1	0	0.481790	-2.458263	3.696642
71	1	0	-1.009981	-2.451386	2.740012
72	6	0	0.209682	-3.985399	-1.053560
73	1	0	0.474300	-4.902261	-1.583794
74	1	0	1.135164	-3.488032	-0.750214
75	16	0	-0.780521	-4.434705	0.453259

Low frequencies --- -3.0528 -1.2633 -0.0007 0.0006 0.0015 4.4401

Low frequencies --- 6.9032 14.7277 25.6349

Zero-point correction= 0.622303 (Hartree/Particle)

Thermal correction to Energy= 0.663244

Thermal correction to Enthalpy= 0.664188

Thermal correction to Gibbs Free Energy= 0.546680

Sum of electronic and zero-point Energies= -2410.276564

Sum of electronic and thermal Energies= -2410.235624

Sum of electronic and thermal Enthalpies= -2410.234680

Sum of electronic and thermal Free Energies= -2410.352188
